# Assessing 24-h movement behaviors in early childhood (0–4 years): Reliability of the My Little Moves app and comparison with accelerometry

**DOI:** 10.1186/s44167-025-00075-x

**Published:** 2025-04-07

**Authors:** Annelinde Lettink, Jelle Arts, Jessica S. Gubbels, Teatske M. Altenburg, Mai J. M. Chinapaw

**Affiliations:** 1 Amsterdam UMC Location Vrije Universiteit Amsterdam, Public and Occupational Health, De Boelelaan 1117, Amsterdam, The Netherlands; 2 Amsterdam Public Health, Health Behaviors and Chronic Diseases, Amsterdam, The Netherlands; 3 Amsterdam Public Health, Methodology, Amsterdam, The Netherlands; 4 Department of Health Promotion, Maastricht University, NUTRIM School of Nutrition and Translational Research in Metabolism, PO Box 616, 6200 MD Maastricht, The Netherlands

**Keywords:** Accelerometer, Time-use diary, Physical activity, Sedentary behavior, Sleep, Reliability, Validity, Infants, Toddlers, Preschoolers

## Abstract

**Background:**

The reliability and validity of proxy-report tools such as the My Little Moves (MLM) app for assessing 24-h movement behaviors (physical activity (PA), sedentary behavior (SB) and sleep) is insufficiently established in early childhood. We aimed to: (1) determine the minimum reporting time (number of hours and days of reported activities) required to reliably assess 24-h movement behaviors of 0–4-year-old children using the MLM app; and (2) evaluate the ability of the MLM app to assess PA, SB, and sleep by hypotheses testing of expected accelerometer-derived accelerations.

**Methods:**

We used data from the MLM study, with at least two days of app data available for 324 children (22.0 ± 12.0 months, 48.3% girls), and accelerometer data (two Axivity AX3 devices, placed on wrist and hip) for 75 children (20.5 ± 11.6 months, 45.3% girls). The Spearman–Brown formula was applied to determine the minimum reporting time needed to achieve a reliability coefficient of 0.70 for time spent in PA, SB and sleep, and 24-h movement behavior compositions. General linear mixed-effects models were used to test our hypothesis that accelerometer-derived acceleration would be lowest during app-based estimates of sleep, followed by SB, and highest during PA. Additionally, we tested 55 sub-hypotheses to examine expected differences and similarities in accelerometer-derived acceleration across app-based activity categories.

**Results:**

The minimum required reporting time was at least 2 days of 20 h for sleep, 2 days of 23 h for PA, 4 days of 17 h for SB, and 2 days of 23 h for the composition of 24-h movement behaviors. As hypothesized, lowest accelerations were recorded during sleep and highest accelerations during PA. We found consistent support for 21 out of 55 sub-hypotheses, with significantly higher acceleration for active play and active transport than for sedentary activities, except for passive transport.

**Conclusions:**

To reliably assess the composition of 24-h movement behaviors in 0–4-year-olds, activities need to be reported in the MLM app for at least two full days. The MLM app shows promise for assessing 24-h movement behaviors, although some specific activities require further investigation.

**Supplementary Information:**

The online version contains supplementary material available at 10.1186/s44167-025-00075-x.

## Background

Healthy 24-h movement behaviors, including physical activity (PA), sedentary behavior (SB), and sleep, are crucial for supporting the growth and development of young children [1–4]. To adequately monitor these behaviors across the age ranges of infants (0–1 year old), toddlers (1–3 years old), and preschoolers (3–5 years old), it is essential to use measurement instruments that are feasible, valid, and reliable, as well as adapted to the child's developmental stage [5].

Accelerometers are widely recognized as a promising method for assessing 24-h movement behaviors, due to their ability to capture body movement data continuously over extended periods of time. While accelerometers are considered valid and reliable for measuring 24-h movement behaviors of preschoolers, school-aged children, and adolescents [6–9], their validity for infants and toddlers remains to be established [9, 10]. Furthermore, there is currently no consensus regarding the optimal accelerometer processing decisions (e.g., choice of cut-points or algorithms to classify PA, SB, or sleep, definition of non-wear time) and measurement protocol (e.g., wear location) for the use of accelerometers in young children [9–11]. Additionally, the current analyses methods do not take into account that accelerometer output in very young children may reflect the movement of others, such as parents carrying their child [12]. Another limitation is the inability of accelerometers to capture contextual information (e.g., social setting and/or location) of different 24-h movement behaviors, restricting our understanding of children’s behaviors and hampering the development of targeted behavioral interventions [13].

Alternatively, proxy-report (often parent-report) tools such as questionnaires or time-use diaries can be used to assess young children's 24-h movement behaviors in a relatively convenient and affordable way. These tools have the additional advantage of the ability to obtain information about the type (e.g., screen use) and context (e.g., location) of the behavior. Therefore, proxy-report tools could potentially provide complementary data to accelerometer data, when used simultaneously [14]. Although a number of proxy-report tools have been developed to assess PA, SB and/or sleep in early childhood, proxy-report tools assessing all 24-h movement behaviors in 0–4-year olds are scarce and generally lack assessment of validity and reliability [15–17]. For this reason, a mobile application (app) was developed to assess 24-h movement behaviors in 0–4-year-old children: the My Little Moves (MLM) app [18]. A content validity study suggested that the MLM app is comprehensive, comprehensible, includes all relevant activity categories and is feasible to complete by parents. Therefore, the MLM app can be considered as a promising tool to proxy-report young children’s 24-h movement behaviors [18].

Unfortunately, proxy-report tools, like the MLM app, have their own limitations such as recall and social desirability bias [14]. Furthermore, the behaviors of young children are often sporadic and intermittent, seldom persisting for continuous periods of time. Consequently, it is very challenging to capture these behaviors accurately. Moreover, most young children are regularly not within the sight of their parents, but for example at daycare or pre-school [19], further contributing to the potential incompleteness of the obtained data. Therefore, before the MLM app can be used in practice, we must first confirm whether the app can be used to obtain an adequate reflection of children’s 24-h movement behaviors.

The absence of a gold standard for assessing 24-h movement behaviors in young children poses a considerable challenge in evaluating the validity of both accelerometers and proxy-report tools such as the MLM app [9, 15, 18]. Additionally, the lack of valid cut-points or algorithms for translating acceleration data into estimates of sleep, SB and PA in infants and toddlers further restricts our ability to evaluate the app's construct validity for assessing all 24-h movement behaviors in the full age range (0–4 years) the app was designed for [9]. Given this challenge in classifying accelerometer data as PA, SB, and sleep, cut‐point‐free accelerometer metrics, such as average acceleration, have been proposed to estimate 24-h movement behaviors [20–23]. To gain insight into the app’s ability to assess 24-h movement behaviors in young children, we tested predetermined hypotheses regarding differences in accelerometer-derived acceleration across app-based estimates of PA, SB, and sleep, i.e., accelerometer-derived acceleration is lowest during sleep, followed by SB, and highest during PA. Additionally, we tested 55 predetermined sub-hypotheses regarding expected differences and similarities in accelerometer-derived acceleration across specific activities assessed in the MLM app.

In this study, our objectives were to (1) determine the minimum reporting time (number of hours and days of reported activities) required to reliably assess 24-h movement behaviors of 0–4-year-old children using the MLM app; and (2) evaluate the ability of the MLM app to assess PA, SB, and sleep by hypotheses testing of expected accelerometer-derived accelerations.

## Methods

### Design and participants

This study is part of “My Little Moves”, a longitudinal observational cohort study. To limit the burden for participants, the “My Little Moves” study was divided into three sub-cohorts. Sub-cohort 1 examined determinants of young children’s 24-h movement behaviors; sub-cohort 2 examined the association between young children’s 24-h movement behaviors and their social-emotional development; and sub-cohort 3 examined the association between young children’s 24-h movement behaviors and their growth and gross motor development. A detailed explanation of the “My Little Moves” study protocol can be found elsewhere [24]. Across all sub-cohorts, data on 24-h movement behaviors were collected by parent-report using the MLM app [18]. Children in sub-cohort 3 additionally wore accelerometers to collect data on 24-h movement behaviors. In the present study, MLM app data from all sub-cohorts were utilized to estimate the minimum number of hours and days required to reliably assess children’s 24-h movement behaviors. MLM app data and accelerometer data of sub-cohort 3 were utilized to test the predefined hypotheses.

Children were included if they were aged 0–4 years, not born extremely premature (< 32 weeks), and had no parent-reported developmental disorders or medical diagnoses that might influence the child’s 24-h movement behaviors or development (e.g., cerebral palsy or developmental language disorder). Additionally, parents were required to have basic Dutch language reading skills and own a smartphone or tablet device. The Medical Ethics Committee of the Amsterdam University Medical Centers approved the study protocol (no. 2022.0020). This study was reported in accordance with the STROBE guidelines for observational studies (Additional file 1) [25].

### Recruitment

Across all cohorts, parents and their children were recruited though early childhood education and care (ECEC) services (e.g., daycare centers) and youth health care services within the Netherlands. These services were approached by email or phone and—in case of agreement—asked to send an information letter to parents through email, newsletters and/or flyers. In addition, researchers were present at ECEC and youth healthcare services to inform parents and answer questions about the study. For sub-cohort 1 and 2, parents and their children were additionally recruited through the dynamic Sarphati Cohort (https://www.sarphaticohort.nl/en/). Parents with children included in the Sarphati Cohort received an information letter through email. Moreover, we recruited parents and children through public spaces such as playgrounds, and community organisations such as sports clubs. Informed consent was obtained from all parents before participating in the study. Informed consent was provided through either a paper form, or online using the survey software Survalyzer (Survalyzer BV, Utrecht, the Netherlands).

### Data collection

#### Procedures

Data were collected between May 2022 and September 2023. After agreeing to participate, parents provided the sex and date of birth of their child on the informed consent form. Additionally, parents provided their own gender identity, age, country of birth, and level of education (high/medium/low) according to ISCED-11 [26], either through previously collected data from the Sarphati Cohort (for parents recruited from the Sarphati cohort), or a brief online survey (for all other parents; Castor EDC, Amsterdam, The Netherlands). In sub-cohort 3, we (AL, JA, or trained assistants) visited the participating children at either their ECEC service or at their parents' home to measure children’s height and weight, and to equip the children with two accelerometers.

#### MLM app

The MLM app is a mobile app designed to be compatible with both smartphones and tablets. The development and content validity evaluation of the MLM app has previously been described by Arts et al. [18]. The app consists of a time-use diary format through which parents can report the activities of their child, using the following activity categories: (1) personal care, (2) eating/drinking, (3) active transport, (4) passive transport, (5) playing, (6) screen use, (7) sitting/lying calmly, (8) sleeping, (9) other activity, (10) I don’t know, and (11) my child was with someone else. Parents reported the activities in 5-min intervals by specifying the start and stop time of the activity. The default duration of the activities was set to 30 min, that could be modified by increasing or decreasing it (in 5-min increments). Consequently, the minimum duration of reported activities is 5 min. Additionally, parents respond to follow-up questions on the intensity (e.g., active or calm play), posture (e.g., lying on the tummy) and context (e.g., location) of the activity. Parents previously indicated that reporting activities in the app takes about 10–30 min per day [18]. Prior to filling in the time-use diary, parents are requested to provide information on their child’s age (i.e., 0–6 months, 6–12 months, 1–2 years, 2–3 years and 3–4 years) and achieved motor milestones (i.e., depending on the child’s age: roll over from back to belly, roll over from belly to back, sit without support, crawl, stand without support, walk without support), along with the corresponding age at which these milestones were reached, if applicable. Based on this information, the content of the app (i.e., activity categories and follow-up questions) is adapted to the developmental stage of the child.

Parents were asked to complete the MLM app for seven consecutive 24-h days from midnight to midnight, without specific instructions regarding requirements for details of reporting (e.g., minimum number of daily reported activities). They received detailed information on how to download and complete the app. In sub-cohort 1 and 2, when parents did not complete the app, they received reminders one week and three weeks after receiving the email with the original request. In sub-cohort 3, parents were instructed to start reporting their child’s activities one day after the placement of the accelerometers. This delay allowed for habituation to wearing the accelerometers. After three days parents received a reminder by email or phone to complete the app, to ensure alignment between the reported activities and accelerometer data.

#### Accelerometers

**Fig. 1:**
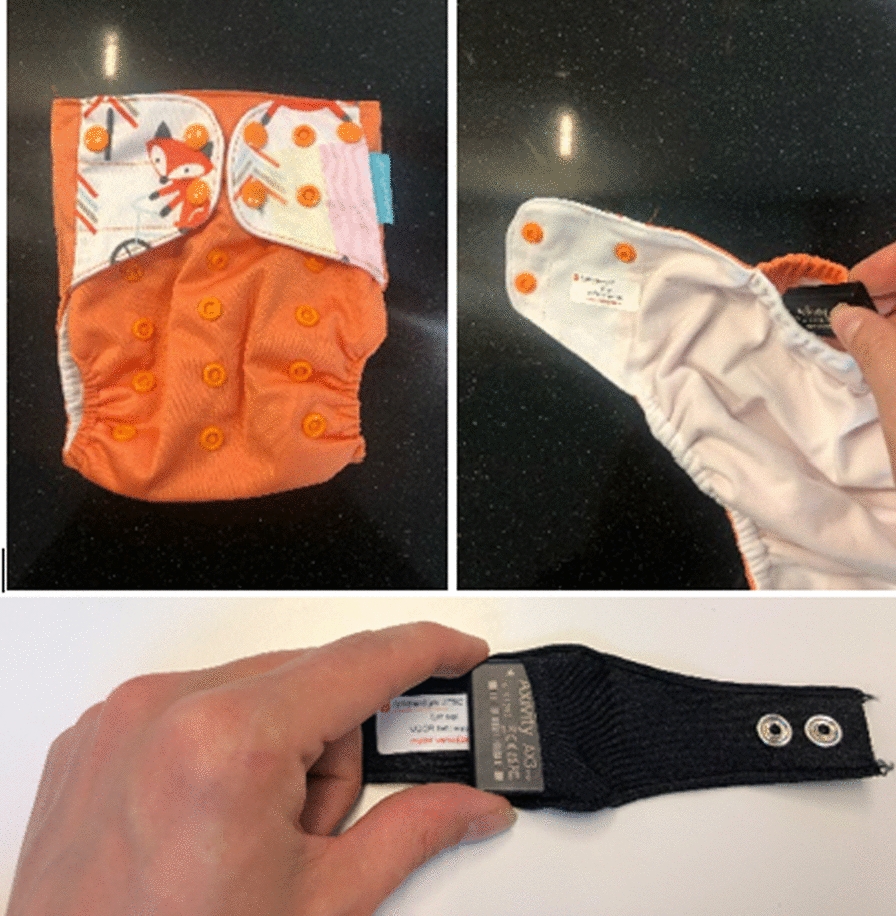
Placement of the Axivity AX3 accelerometers in the diaper pants (upper panel) and wristband (lower panel)

Axivity AX3 (Axivity, Newcastle, UK) accelerometers are small (23 × 32.5×7.6 mm) and lightweight (11 g) devices that capture raw triaxial acceleration values in gravitational units (*g*). Accelerometers were initialized (sampling frequency of 50 Hz; dynamic range of ± 8 *g*) and data were downloaded using OMGUI open-source software (V43, Open Movement, Newcastle University, UK). Accelerometers were placed on the child’s left wrist (using wristbands [27]) and on the right hip (incorporated in diaper/short tight pants), see Fig. 1. Subsequently, parents were asked to let their child wear these accelerometers for 8 consecutive days, 24 h per day, except during water activities such as showering or bathing. Parents received detailed instructions regarding placement of the devices and were asked to reapply the pants and wristband after removal.

#### Growth

Height and weight were measured to calculate the body mass index (BMI) z-score. Height was measured to the nearest 0.1 cm using a portable stadiometer (Marsden HM-250P) or a length board (Seca 417) depending on whether the child could stand or not, respectively. Weight was measured to the nearest 0.1 kg using a calibrated electronic (baby) scale (Seca 354). Children were lightly dressed and barefoot during these measurements. Subsequently, we used the open-source R package anthro developed by the WHO to compute BMI z-scores, based on the child’s height, weight, sex, and age [28].

### Data preprocessing

#### MLM app data

MLM app data were downloaded and provided as.json files by the software developer. We converted these files into.csv format, structuring each row to represent an individual activity entry along with the corresponding responses to follow-up questions. Subsequently, we calculated the frequency and time spent and in all activity categories for each participant per day. In addition, time spent in PA, SB and sleep was calculated based on the activity categories and the follow-up question in the app on the posture of the activity. Table 1 shows how we classified these 24-h movement behaviors from the activity categories and follow-up questions in the app. The categories “I don’t know”, “my child was with someone else”, and “other activity” were excluded from the analyses.

**Table 1:** Classification of physical activity, sedentary behavior and sleep based on the (developmentally appropriate) activity categories and posture as reported in the My Little Moves app

Activity category	Developmental group	Posture	24-h movement behavior
Personal care (e.g., having a diaper changed or showering)	All	NA	SB
Eating/drinking (e.g., being breastfed or eating dinner)	All	NA	SB
Sitting/lying calmly (e.g., lying or sitting on a couch)	0–1 year	Being carried	SB
		Lying on tummy	PA
		Lying on back	SB
		Lying on side	SB
		Sitting with support	SB
	0–1 year & milestone ‘sitting without support’ achieved	Sitting without support	PA
	> 1 year	Lying	SB
		Sitting	SB
	All	Changing posture	SB
		I don’t know	SB
Playing: Calm/I don’t know (e.g., reading a book or drawing)	0–1 year	Being carried	SB
		Lying on tummy	PA
		Lying on back	SB
		Lying on side	SB
		Sitting with support	SB
	0–1 year & milestone ‘sitting without support’ achieved	Sitting without support	PA
	> 1 year	Lying	SB
		Sitting	SB
	< 2 years	Standing with support	SB
	6 months–2 years & milestone ‘standing without support’ achieved	Standing without support	PA
	> 2 years	Standing	SB
	All	Changing posture	SB
		I don’t know	SB
Playing: Active (e.g., crawling or running in a playground)	0–1 year	Being carried	PA
		Lying on tummy	PA
		Lying on back	PA
		Lying on side	PA
		Sitting with support	PA
	0–1 year & milestone ‘sitting without support’ achieved	Sitting without support	PA
	> 1 year	Lying	PA
		Sitting	PA
	< 2 years	Standing with support	PA
	6 months–2 years & milestone ‘standing without support’ achieved	Standing without support	PA
	> 2 years	Standing	PA
	All	Changing posture	PA
	All	I don’t know	PA
Passive transport (e.g., sitting in a car or bicycle seat)	All	NA	SB
Sleeping	All	NA	Sleep
Screen use: Watching/Calm play/I don’t know (e.g., watching or scrolling on a tablet)	All	NA	SB
Screen use: Active play (e.g., dancing or playing active games in front of a television)	> 6 months	NA	PA
Active transport (e.g., walking or cycling to the supermarket)	> 2 years or milestone ‘walking without support’ achieved	NA	PA

Abbreviations: *NA* not applicable, *PA* Physical activity, *SB* sedentary behavior

The time spent in each of the 24-h movement behaviors is mutually exclusive, as time spent on one behavior reduces the time available for the other behaviors. Compositional data analysis is a statistical technique that accounts for the mutually exclusive nature of 24-h movement behaviors by analyzing the relative proportions of time spent in each behavior rather than their absolute values [29–31]. Therefore, we transformed time spent in PA, SB and sleep into a 3-part composition, containing three pairs of coordinates using the isometric log-ratio (ilr) method [30, 32, 33] using the R package robCompositions [34]. Each pair of coordinates encompasses a distinct behavior relative to all other remaining behaviors, for instance, when PA serves as the reference: ilr.PA represents PA relative to SB and sleep, and ilr.PA2 represents SB relative to sleep:

ilr.PA = $$\sqrt{\frac{2}{3}}$$ $$\text{ln}(\frac{physical\, activity}{{(sedentary\, behavior*sleep)}^\frac{1}{2}})$$

ilr.PA2 = $$\sqrt{\frac{1}{2}}$$ $$\text{ln}(\frac{sedentary\, behavior}{sleep})$$

Note that we only included the first pivot coordinate of each pair in the analyses (i.e., ilr.PA, ilr.SB and ilr.sleep). This coordinate best reflects the movement behavior composition and can be interpreted as the relative importance of one behavior with respect to the remaining behaviors, while the second coordinate does not contain all behaviors [35].

#### Accelerometer data

Accelerometer files from both hip and wrist placements were processed using the open-source R package GGIR version 2.9-0 [19]. Signal processing included auto-calibration using local gravity as a reference [36], detecting sustained abnormally high values, and non-wear detection [37]. We calculated aggregated outcome values over 5-s epochs for the most commonly used metrics: vector magnitude of acceleration corrected for gravity (Euclidian norm minus one, ENMO) and the mean amplitude deviation (MAD) [19, 38, 39]. Accelerometer files were excluded if post-calibration error was greater than 0.01 *g* [36] or if parents indicated that the wrist accelerometer was worn on the child’s right instead of the left wrist (n = 2). Additionally, non-wear periods in the accelerometer data were excluded from further analyses.

#### Matching of parent-reported activity categories with accelerometer data

**Fig. 2:**
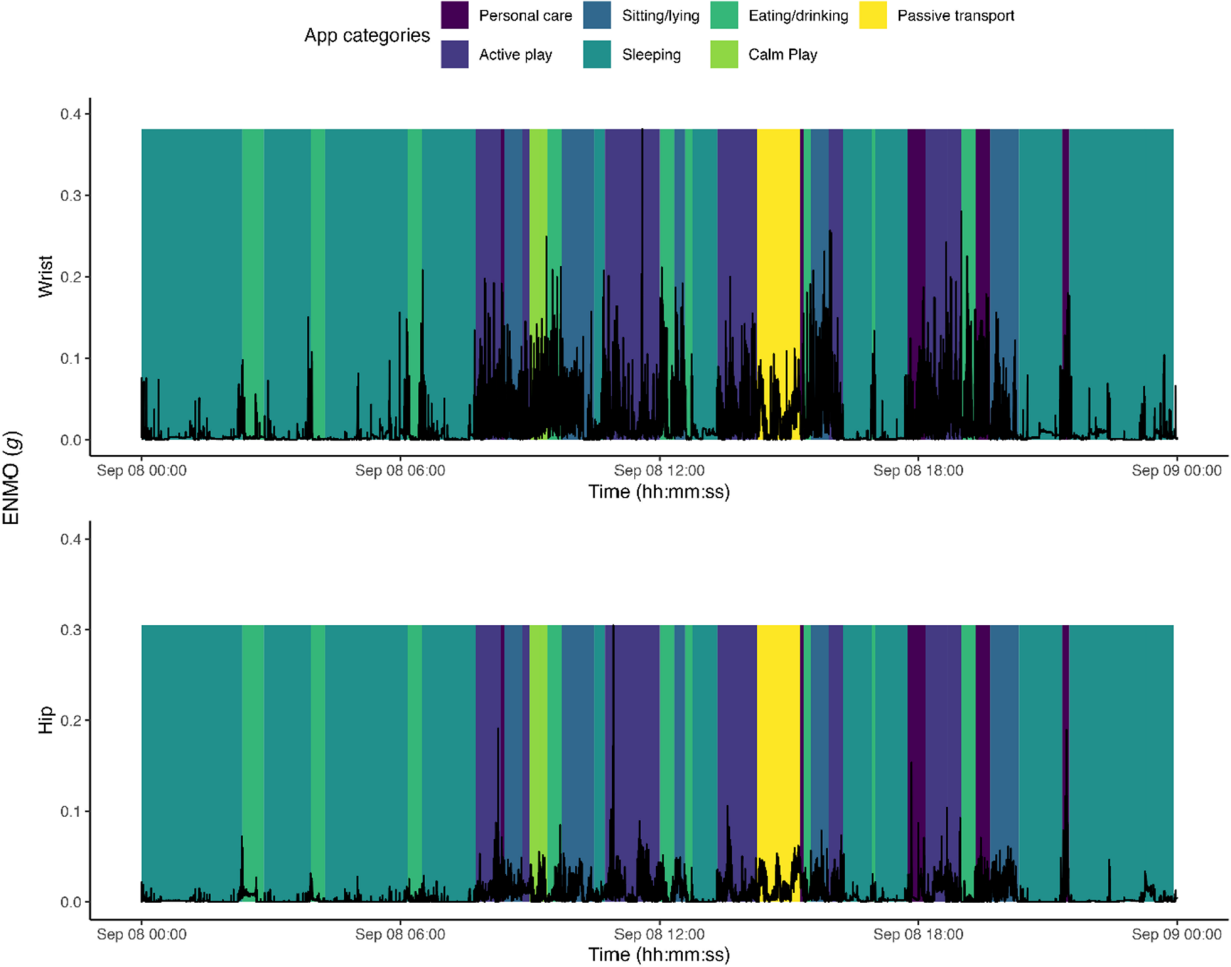
Example of synchronization of time-stamped data from both the My Little Moves (MLM) app and accelerometers of one parent–child dyad over one day

The start and end times of all parent-reported activity categories in the MLM app were synchronized with acceleration signal time series from the child’s left wrist (Fig. 2 upper panel) and right hip (Fig. 2 lower panel). Figure 2 illustrates an example of this data synchronization process for one parent–child dyad. We matched all 5-s epochs greater than or equal to the start time of the reported activity category and less than or equal to the reported end time. As acceleration distributions were positively skewed, for both hip and wrist data, we calculated median ENMO and MAD for each reported activity category. By aligning the time-stamped app data with the time-stamped acceleration data, we could calculate and directly compare the median acceleration values across app-based activity categories and the corresponding 24-h movement behaviors. Descriptive analyses were conducted to examine the duration, frequency, and the distribution of accelerations related to the different app-based 24-h movement behaviors and activity categories.

### Statistical analyses

All statistical analyses were conducted in R (version 4.1.2) and considered at the 2-tailed α level of .05. Our R code can be found in our GitHub repository [40]. We calculated the characteristics of all participants included in the reliability and comparative analyses. Additionally, we calculated median and inter quartile range [25th–75th percentile] of duration and acceleration, as well as frequency and percentage of app-based 24-h movement behaviors and activity categories.

#### Minimum reporting time required for reliable estimates of 24-h movement behaviors

Normality of all data was checked by visually inspecting the histograms and Q-Q plots, which revealed non-normal distributions for the MLM app-based estimates of time spent in PA, SB, and sleep (min/day). To address this, we applied a log-transformation to these estimates.

We assessed differences between weekdays and weekend days in time spent in PA, SB, and sleep (min/day), and the 3-part composition (ilr.PA, ilr.SB, and ilr.sleep) by fitting separate general linear mixed-effects models (GLMMs) adjusted for the child’s sex and age to determine the need for inclusion of a weekend day. For this analysis, we included participants with a reporting time of at least two days of ≥ 12 h, including at least one weekend day. We assessed the differences for each hourly increment from 12 up to 24 h.

To determine the minimum number of days required to reach reliability coefficients of 0.70, we used the Spearman–Brown prophecy formula inputting single-day intra-class correlations (ICCs) [41]. Single-day ICCs were calculated using two-way mixed effects, absolute agreement, single measurement models [42] using the R package psych [43]. We calculated ICCs for all potential combinations of inclusion criteria (i.e., minimum hours of reported activities per day), ranging from a minimum of 12 to a maximum of 24 h of data, across periods of two to seven days. For participants with data exceeding the required number of days, we randomly selected the days used to compute the ICC. For example, to calculate the ICCs for two days of data, we randomly selected two days for participants with more than two valid days of data (with the same approach for three to seven days). This process was repeated five times, and the average ICC per criterion is presented. This method is used to mitigate potential selection biases from choosing specific days for inclusion in reliability analyses, allowing for a more accurate estimate of day-to-day variability and thereby a more robust reliability estimate [44–46].

#### Comparison of MLM app-based estimates with accelerometry

**Fig. 3:**
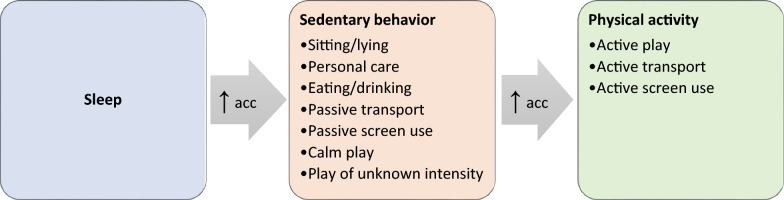
Hypothesized differences and similarities in accelerometer-derived acceleration (acc) across activity categories and 24-h movement behaviors assessed using the My Little Moves (MLM) app. Similar accelerometer-derived accelerations were expected within app-based SB activity categories (orange; sub-hypotheses 1–21) and within app-based PA categories (green; sub-hypotheses 22–24). Lowest accelerometer-derived accelerations were expected during app-based sleep (blue), followed by app-based SB activity categories (orange), and highest accelerometer-derived accelerations were expected during app-based PA categories (green) (sub-hypotheses 25–55)

To evaluate the ability of the MLM app to assess PA, SB, and sleep, we formulated hypotheses for both app-based 24-h movement behaviors and activity categories regarding expected accelerometer-derived accelerations. First, for 24-h movement behaviors, we hypothesized that accelerometer-derived acceleration would be lowest during app-based sleep, followed by app-based SB, and that accelerometer-derived acceleration would be highest during app-based PA. Second, for the app-based activity categories (Table 1), we formulated 55 sub-hypotheses, which are also illustrated in Fig. 3:

- Sub-hypotheses 1–21: We hypothesized that accelerometer-derived acceleration would be similar (i.e., not significantly different) across the seven app-based activity categories predominantly classified as SB (i.e., including sitting/lying, personal care, eating/drinking, passive transport, passive screen use, calm play, and play of unknown intensity).
- Sub-hypotheses 22–24: We hypothesized that accelerometer-derived acceleration would be similar (i.e., not significantly different) across the three app-based activity categories predominantly classified as PA (i.e., including active transport, active play, and active screen use (e.g., dancing in front a television)).
- Sub-hypotheses 25–55: We hypothesized that accelerometer-derived acceleration would be lowest during the app-based activity category sleep, followed by the seven app-based activity categories predominantly classified as SB, and that accelerometer-derived acceleration would be highest during the three app-based activity categories predominantly classified as PA (see Fig. 3).

To test these hypotheses, for the different app-based 24-h movement behaviors as well as activity categories we compared accelerations by fitting four separate GLMMs for each acceleration metric (i.e., ENMO hip, ENMO wrist, MAD hip, and MAD wrist). Non-normality of the residuals of the model was confirmed by visual inspection of the residuals plot. Therefore, models were reported for log-transformed acceleration metrics. Significance was determined using Satterthwaite’s method to estimate degrees of freedom and generate p-values for mixed models [47].

Finally, we aimed to examine variations in acceleration across the various postures reported within activity categories in the MLM app (e.g., lying on the tummy vs. lying on the back). However, due to low variety in reported postures within the activity categories this was not possible (see Results Sect. "Descriptive statistics").

##### Main hypothesis: differences in accelerometer-derived acceleration between app-based 24-h movement behaviors

We examined differences in acceleration between the app-based 24-h movement behaviors by fitting four GLMMs for the acceleration metrics (i.e., ENMO hip, ENMO wrist, MAD hip, and MAD wrist). These models incorporated a fixed effect for the 24-h movement behavior and nested random effects to account for repeated measures of participants across these behaviors. Additionally, these models were adjusted for the child’s sex and age. The explained variance (R-squared values) of the fixed effects were defined as very weak (.00–.02), weak (.02–0.13), moderate (.13–0.26), and substantial (> .26) using Cohen’s recommendations [48].

##### Sub-hypotheses 1–55: similarities in acceleration across app-based activity categories classified as SB (1–21) or PA (22–24), and differences in acceleration between app-based activity categories classified as SB, PA and sleep (25–55)

We fitted GLMMs for the acceleration metrics (i.e., ENMO hip, ENMO wrist, MAD hip, and MAD wrist) incorporating a fixed effect for the respective activity category and nested random effects to account for multiple measurements across these categories. Additionally, these models were adjusted for the child’s sex and age.

## Results

### Participants characteristics

**Fig. 4:**
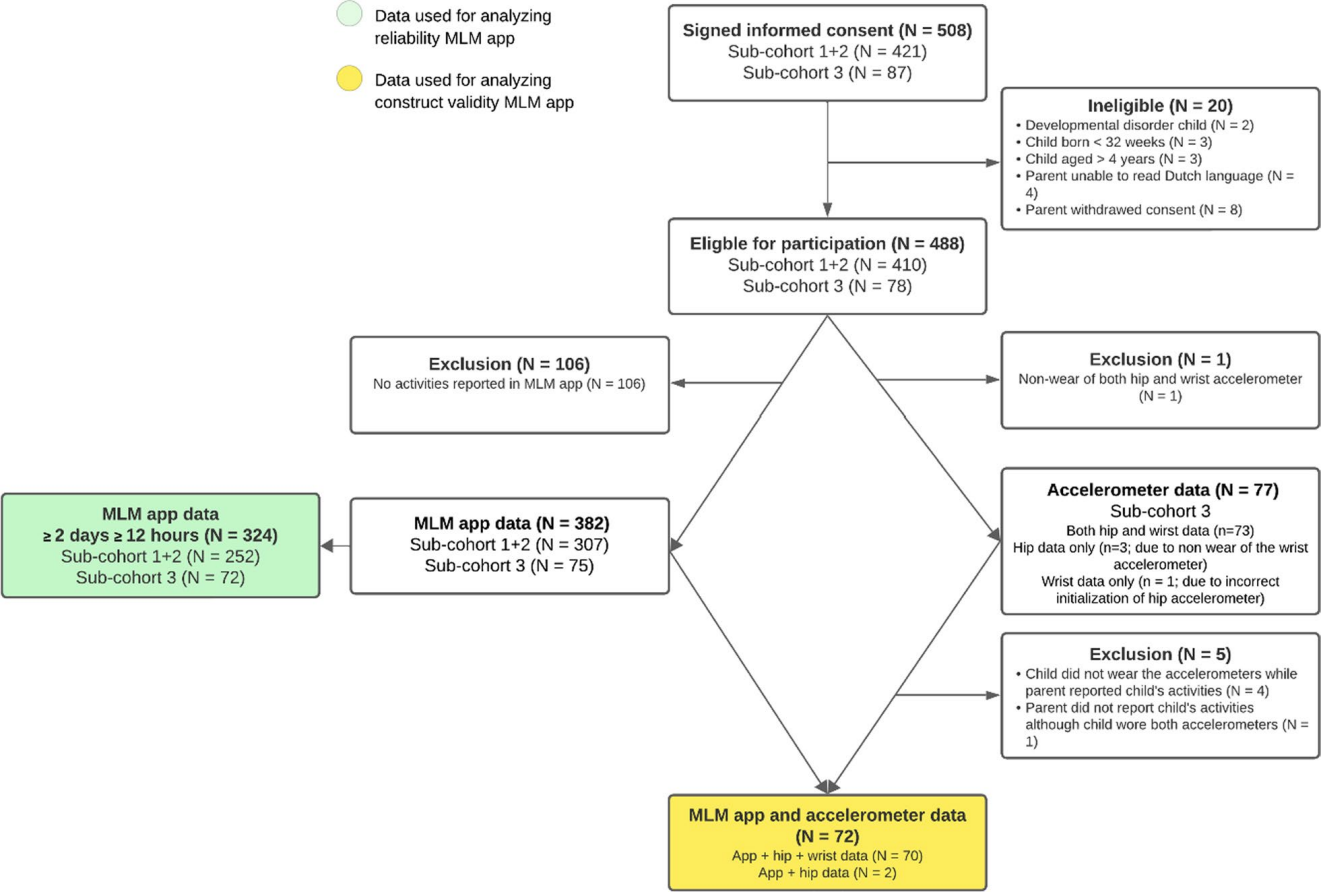
Flowchart of the recruitment process for participants of this study

Figure 4 presents the flowchart of the recruitment process for participation in this study. Across all sub-cohorts, 508 children were enrolled, of which 488 parents and children met the inclusion criteria for the present study. In total, parents of 382 children reported activities in the MLM app. In sub-cohort 3, 77 out of 78 children wore at least one accelerometer, although for five of these children, parents did not report any activities in the MLM app. Consequently, we have both MLM app and accelerometer data for 72 children, with complete data (i.e., both hip and wrist accelerometer data) for 70 children and only hip accelerometer data for 2 children. Table 2 presents characteristics of participating children and their parents.

**Table 2:** Characteristics of participating children and their parents

	Reliability MLM app		Comparison of MLM app-based estimates with accelerometry	
	Parents^a^	Children	Parents	Children
N	317	324	69	72
Age (M ± SD)	36.2 ± 4.2 years ^b^	22.0 ± 12.0 months	36.0 ± 5.1 years ^b^	20.2 ± 11.2 months
Sex (% female)	288 (91.1) ^c^	158 (48.8)	61 (88.4) ^c^	33 (45.8)
BMI z-score (M ± SD)	–	–	–	0.31 ± 1.20
Country of birth mother Netherlands/Other	227/63	–	47/16	–
Country of birth father Netherlands/Other	227/54	–	50/13	–
Level of education parent High/Medium/Low	238/25/4	–	55/8/0	–

^a^Seven parents participated with two children

^b^Missing data for age parent, 44 for reliability, and 7 for comparison with accelerometry

^c^Missing data for sex parent, 1 for reliability and 0 for comparison with accelerometry

### Minimum reporting time required for reliable estimates of 24-h movement behaviors

Out of 382 parents who filled in the MLM app, 324 (84.8%) parents met the criterion of reporting ≥ 12 h of activities on at least two days, including at least one weekend day (Fig. 4). Depending on the minimum number of hours reported in the app, there was a significant difference between week and weekend days for time spent in different 24-h movement behaviors (see Table 1 in Additional file 2). Specifically, significant differences in PA, SB, ilr.PA, ilr.SB and ilr.sleep were observed between week and weekend days with valid day criteria up to 17, 16, 18, 17, and 18 h of data, respectively. Additionally, significant differences in sleep were observed between weekdays and weekend days for all criteria, except for ≥ 16, ≥ 17, and ≥ 19 h of data.

Table 3 presents the ICCs and corresponding minimum number of days of reporting time required to achieve a reliability of 0.70 for app-based estimates of PA, SB, sleep, and compositions of 24-h movement behaviors. Single day ICCs ranged from 0.15 to 0.57 for PA, 0.21 to 0.42 for SB, 0.28 to 0.55 for sleep, 0.20 to 0.56 for ilr.PA, 0.26 to 0.54 for ilr.SB and 0.17 to 0.57 for ilr.sleep. When focusing the highest percentage of participants meeting the required reporting time to reach a reliability of 0.70, a minimum of 2 days of ≥ 23 h was required for PA, with 83.3% of the participants meeting this criterion. SB required a minimum of 4 days of ≥ 17 h (66.7% of participants), and sleep required at least of 2 days of ≥ 20 h (92.3% of participants). Regarding the composition of 24-h movement behaviors, both ilr.PA and ilr.sleep required at least 2 days of 22 h of app reporting time (87.7% of participants) and ilr.SB required at least 2 days of ≥ 23 h (83.3% of participants).

**Table 3:** Number of days of reporting required for reliable My Little Moves app-based estimates of 24-h movement behaviors

Outcome	Minimum reporting time (hours/day)	Single day ICC [95% CI]^a^	Minimum days required to achieve reliability of 70%	Number (N (%)) parents meeting minimum reporting time
PA (min/day)	≥ 12	0.16 [0.10;0.23]	11.96	–
	≥ 13	0.15 [0.09;0.22]	13.12	–
	≥ 14	0.15 [0.09;0.22]	13.33	–
	≥ 15	0.22 [0.15;0.29]	8.48	–
	≥ 16	0.35 [0.26;0.44]	4.43	157 (48.46)
	≥ 17	0.38 [0.29;0.48]	3.75	216 (66.67)
	≥ 18	0.39 [0.30;0.49]	3.62	204 (62.96)
	≥ 19	0.42 [0.32;0.52]	3.20	193 (59.57)
	≥ 20	0.46 [0.37;0.55]	2.73	250 (77.16)
	≥ 21	0.49 [0.39;0.59]	2.41	242 (74.69)
	≥ 22	0.53 [0.43;0.63]	2.06	226 (69.75)
	**≥ 23**	**0.57 [0.46;0.66]**	**1.79**	**270 (83.33)**
	24	0.52 [0.38;0.67]	2.18	160 (49.38)
SB (min/day)	≥ 12	0.21 [0.15;0.27]	8.85	–
	≥ 13	0.26 [0.19;0.32]	6.80	199 (61.42)
	≥ 14	0.25 [0.18;0.32]	7.16	–
	≥ 15	0.30 [0.23;0.38]	5.41	168 (51.85)
	≥ 16	0.36 [0.27;0.45]	4.23	157 (48.46)
	** ≥ 17**	**0.38 [0.30;0.48]**	**3.74**	**216 (66.67)**
	≥ 18	0.38 [0.29;0.48]	3.74	204 (62.96)
	≥ 19	0.40 [0.31;0.51]	3.46	193 (59.56)
	≥ 20	0.42 [0.32;0.53]	3.25	179 (55.25)
	≥ 21	0.40 [0.30;0.52]	3.46	161 (49.69)
	≥ 22	0.41 [0.30;0.53]	3.38	150 (46.30)
	≥ 23	0.38 [0.27;0.52]	3.80	125 (38.58)
	24	0.36 [0.23;0.54]	4.20	48 (14.81)
Sleep (min/day)	≥ 12	0.28 [0.22;0.35]	5.89	260 (80.25)
	≥ 13	0.30 [0.24;0.37]	5.35	252 (77.78)
	≥ 14	0.32 [0.26;0.39]	4.88	274 (84.57)
	≥ 15	0.36 [0.29;0.44]	4.14	231 (71.30)
	≥ 16	0.40 [0.31;0.49]	3.52	232 (71.60)
	≥ 17	0.46 [0.38;0.56]	2.69	279 (86.11)
	≥ 18	0.51 [0.42;0.60]	2.25	268 (82.72)
	≥ 19	0.54 [0.44;0.63]	2.02	266 (82.10)
	**≥ 20**	**0.55 [0.45;0.65]**	**1.92**	**299 (92.28)**
	≥ 21	0.54 [0.44;0.65]	1.95	293 (90.43)
	≥ 22	0.55 [0.45;0.66]	1.88	284 (87.65)
	≥ 23	0.54 [0.42;0.66]	1.99	270 (83.33)
	24	0.50 [0.36;0.66]	2.37	160 (49.38)
ilr.PA	≥ 12	0.20 [0.14;0.27]	9.29	–
	≥ 13	0.20 [0.13;0.26]	9.60	–
	≥ 14	0.21 [0.14;0.28]	9.00	–
	≥ 15	0.27 [0.19;0.35]	6.41	94 (29.01)
	≥ 16	0.39 [0.31;0.49]	3.58	232 (71.60)
	≥ 17	0.41 [0.32;0.50]	3.40	216 (66.67)
	≥ 18	0.41 [0.32;0.51]	3.31	204 (62.96)
	≥ 19	0.43 [0.34;0.53]	3.04	193 (59.56)
	≥ 20	0.41 [0.32;0.50]	2.32	250 (77.16)
	≥ 21	0.52 [0.42;0.62]	2.14	242 (74.69)
	**≥ 22**	**0.56 [0.46;0.66]**	**1.83**	**284 (87.65)**
	≥ 23	0.58 [0.47;0.68]	1.72	270 (83.33)
	24	0.53 [0.39;0.68]	2.11	160 (49.38)
ilr.SB	≥ 12	0.26 [0.19;0.33]	6.71	210 (64.81)
	≥ 13	0.27 [0.21;0.33]	6.43	199 (61.42)
	≥ 14	0.28 [21;0.35]	6.09	170 (52.47)
	≥ 15	0.32 [0.25;0.40]	4.91	231 (71.30)
	≥ 16	0.40 [0.31;0.49]	3.54	232 (71.60)
	≥ 17	0.42 [0.33;0.51]	3.23	216 (66.67)
	≥ 18	0.44 [0.34;0.53]	3.02	204 (62.96)
	≥ 19	0.45 [0.35;0.55]	2.87	266 (82.10)
	≥ 20	0.48 [0.38;0.58]	2.54	250 (77.16)
	≥ 21	0.49 [0.38;0.60]	2.42	242 (74.69)
	≥ 22	0.53 [0.43;0.64]	2.03	226 (69.75)
	** ≥ 23**	**0.54 [0.43;0.65]**	**1.98**	**270 (83.33)**
	24	0.49 [0.36;0.66]	2.40	160 (49.38)
ilr.sleep	≥ 12	0.17 [0.11;0.24]	11.13	–
	≥ 13	0.18 [0.11;0.24]	10.88	–
	≥ 14	0.17 [0.11;0.24]	11.47	–
	≥ 15	0.22 [0.14;0.30]	8.43	–
	≥ 16	0.37 [0.28;0.46]	3.97	232 (71.60)
	≥ 17	0.40 [0.31;0.49]	3.55	216 (66.67)
	≥ 18	0.39 [0.30;0.49]	3.61	204 (62.96)
	≥ 19	0.43 [0.33;0.53]	3.13	193 (59.57)
	≥ 20	0.49 [0.40;0.58]	2.44	250 (77.16)
	≥ 21	0.51 [0.41;0.60]	2.23	242 (74.69)
	**≥ 22**	**0.54 [0.44;0.63]**	**1.98**	**284 (87.65)**
	≥ 23	0.57 [0.47;0.67]	1.74	270 (83.33)
	24	0.54 [0.40;0.68]	2.03	160 (49.38)

^a^Two-way random intra-class correlation coefficient, consistency, single

The results corresponding to the highest percentage participants meeting the required reporting time is highlighted in bold

Abbreviations: *CI* confidence interval, *ENMO* Euclidean norm minus one, *ICC* intra-class correlation coefficient, *ilr* isometric log ratio, *MAD* mean amplitude deviation, *MLM* app My Little Moves app, *PA* physical activity, *SB* sedentary behavior

### Comparison of MLM app-based estimates with accelerometry

#### Descriptive statistics

Duration, frequency and accelerometer-derived acceleration for all app-based 24-h movement behaviors, activities, and postures are presented in Tables 2 and 7 in Additional file 2. A minor proportion (3.4%) of all reported activities fell in the categories “I don’t know”, “my child was with someone else”, and “other activity”. The median reported duration was longest for the category “my child was with someone else” (510 min), which on average was reported at least once on 37.1% of all measurement days. Regarding 24-h movement behaviors, activities classified as SB were most frequently reported (62.9%), whereas activities classified as PA (16.9%) and sleep (16.8%) were reported equally often. Median duration per reported activity was longest for sleep (220 min), followed by activities classified as PA (30 min) and SB (25 min).

For specific activity categories, playing was most frequently reported (23.4%), of which the majority was active play (14.3%), followed by calm play (8.9%) and a negligible portion of play of unknown intensity (0.3%). Other commonly reported activities included eating/drinking (19.8%), sleeping (16.8%), personal care (14.4%), and passive transport (11.1%), while sitting/lying (7.1%), active transport (2.1%), and screen use (2.0%) represent a small proportion of the reported activities. Nearly all reported screen use was passive, with active screen use reported only three times. The median duration was longest for the category sleeping (220 min). For all other activity categories, the median durations ranged from 15 min (personal care) to 60 min (play of unknown intensity).

Regarding reported postures within activity categories, changing posture was reported most frequently (27.0%, 55.8%, and 65.4% for sitting/lying, calm play and active play, respectively). Some postures were rarely reported. For example, within the category sitting/lying, lying on the side was reported only five times (0.8%), sitting without support nine times (1.3%), and lying on the tummy eleven times (1.7%). Within active play, being carried and lying on the side were not reported at all.

#### Main hypothesis: differences in accelerometer-derived acceleration between app-based 24-h movement behaviors

**Fig. 5:**
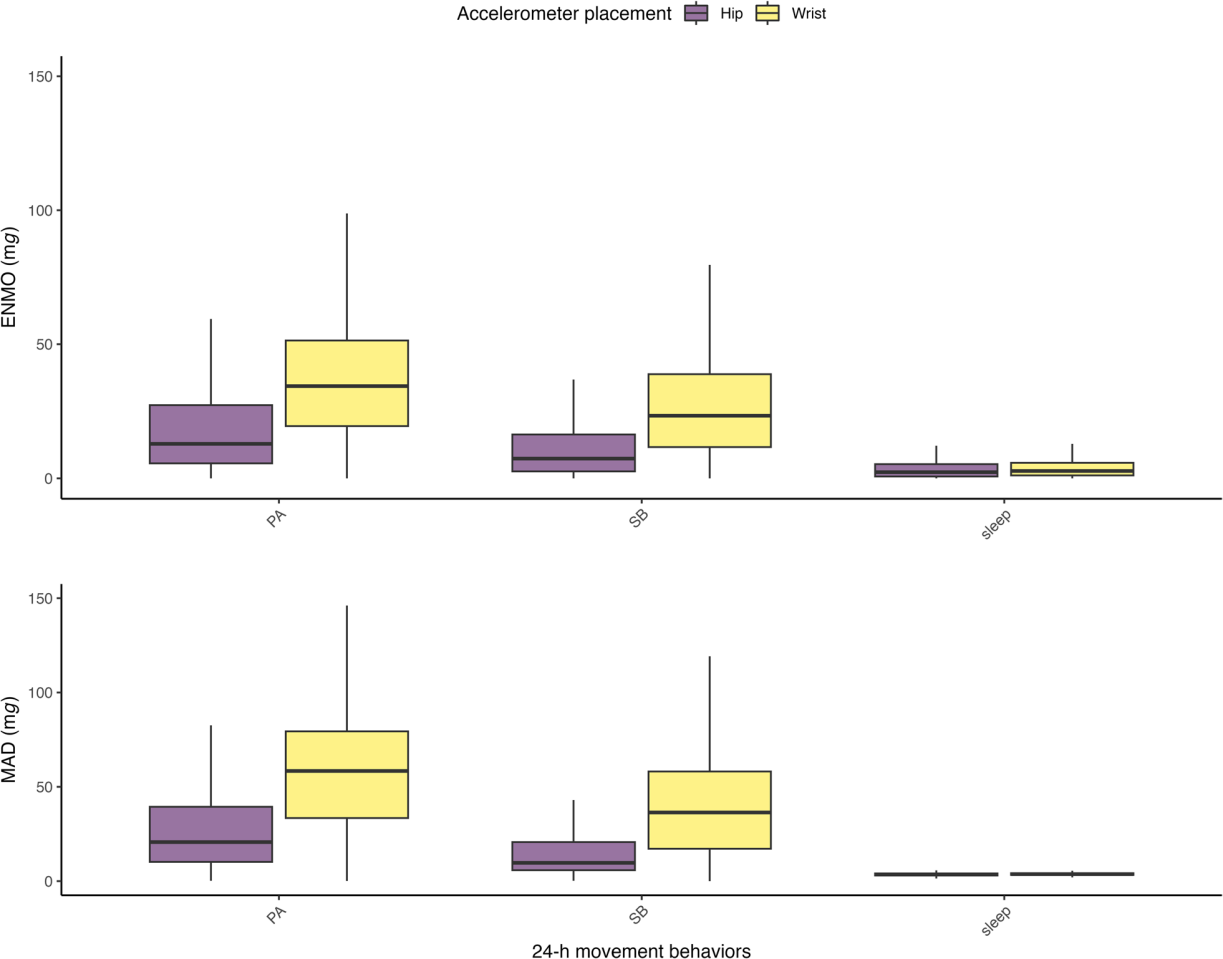
Euclidean norm minus one (ENMO) and mean amplitude deviation (MAD) acceleration of both hip and wrist accelerometers for physical activity (PA), sedentary behavior (SB) and sleep assessed by the My Little Moves app

The complete model outputs of the GLMMs are presented in Additional file 2. Table 4 outlines the consistency of the findings regarding the hypothesized differences and similarities in accelerometer-derived acceleration between app-based 24-h movement behaviors and app-based activity categories, indicating consistent support (i.e., for both metrics and accelerometer placements), inconsistent support (i.e., for some metrics and accelerometer placements), and lack of support for the hypotheses. Figure 5 presents the distribution of acceleration during the app-based 24-h movement behaviors, including median and quartiles. As hypothesized, within both placements and metrics, acceleration significantly differed between the app-based 24-h movement behaviors, with lowest acceleration observed during sleep*,* followed by SB and highest acceleration observed during PA (*p* < 0.001). For hip acceleration, the explained variance of 24-h movement behaviors ranged from moderate (ENMO *R*^*2*^ = .14) to substantial (MAD *R*^*2*^ = .28), while for wrist acceleration the explained variance was substantial for both metrics (ENMO *R*^*2*^ = .28, MAD *R*^*2*^ = .37).

**Table 4:** Hypotheses testing of anticipated differences and similarities in accelerometer-derived acceleration between app-based 24-h movement behaviors and app-based activity categories

Hypotheses	Consistently ( +), inconsistently ( ±), not (−) supported *
Differences between 24-h movement behaviors	+ (3), ± (0),− (0)**
Sleep < Sedentary behavior	+
Sleep < Physical activity	+
Sedentary behavior < Physical activity	+
**Similarities across activity categories classified as SB**	+ (2), ± (8),− (11)**
Passive transport = Calm play	± (wrist ENMO) ***
Passive transport = Play of unknown intensity	± (wrist ENMO and MAD)
Passive transport = Sitting/lying	–
Passive transport = Eating/drinking	–
Passive transport = Personal care	–
Passive transport = Passive screen use	–
Passive transport = Sitting/lying	–
Passive screen use = Play of unknown intensity	± (hip MAD)
Passive screen use = Sitting/lying	–
Passive screen use = Eating/drinking,	–
Passive screen use = Personal care	–
Passive screen use = Calm play	–
Sitting/lying = Eating/drinking	+
Sitting/lying = Personal care	± (hip MAD, wrist ENMO and MAD)
Sitting/lying = Play of unknown intensity	± (hip ENMO and MAD)
Sitting/lying = Calm play	–
Eating/drinking = Play of unknown intensity	± (hip ENMO and MAD, and wrist ENMO)
Eating/drinking = Calm play	–
Personal care = Eating/drinking	± (wrist ENMO and MAD)
Personal care = Play of unknown intensity	± (hip ENMO and MAD, wrist ENMO)
Calm play = Play of unknown intensity	+
**Similarities across activity categories classified as PA**	+ (0), ± (3),− (0)**
Active transport = Active screen use	± (wrist ENMO and MAD)
Active play = Active transport	± (wrist ENMO and MAD)
Active play = Active screen use	± (hip ENMO, wrist ENMO and MAD)
**Differences between activity categories**	+ (19), ± (4), − (8)**
Sleep < Sitting/lying	+
Sleep < Personal care	+
Sleep < Eating/drinking	+
Sleep < Passive screen use	+
Sleep < Passive transport	+
Sleep < Active transport	+
Sleep < Active play	+
Sleep < Calm play	+
Sleep < Play of unknown intensity	+
Sleep < Active screen use	± (wrist ENMO and MAD)
Active play > Sitting/lying	+
Active play > Eating/drinking	+
Active play > Personal care	+
Active play > Passive screen use	+
Active play > Calm play	+
Active play > Play of unknown intensity	± (hip MAD)
Active play > Passive transport	–
Active transport > Sitting/lying	+
Active transport > Eating/drinking	+
Active transport > Personal care	+
Active transport > Passive screen use	+
Active transport > Calm play	+
Active transport > Play of unknown intensity	± (hip ENMO and MAD)
Active transport > Passive transport	± (hip MAD)
Active screen use > Sitting/lying	–
Active screen use > Eating/drinking	–
Active screen use > Personal care	–
Active screen use > Passive screen use	–
Active screen use > Passive transport	–
Active screen use > Calm play	–
Active screen use > Play of unknown intensity	–

*Hypotheses consistently supported by both ENMO and MAD acceleration data, across both wrist and hip placements, are denoted with (+). Hypotheses that are inconsistently supported—i.e., when support was found, but not by both ENMO and MAD acceleration data, across both wrist and hip placements—are denoted with (±). Hypotheses consistently not supported by either ENMO or MAD data, or at neither wrist nor hip placement, are denoted with (−)

**Number of hypotheses that are consistently (+), inconsistently (±), and not (−) supported

***For hypotheses inconsistently supported (±), the specific accelerometer metrics and placements supporting the hypothesis are presented

*Abbreviations: ENMO* Euclidean norm minus one, *MAD* mean amplitude deviation, *PA* physical activity, *SB* sedentary behavior

#### Sub-hypotheses 1–21: similarities in accelerometer-derived acceleration across app-based activity categories classified as SB

**Fig. 6:**
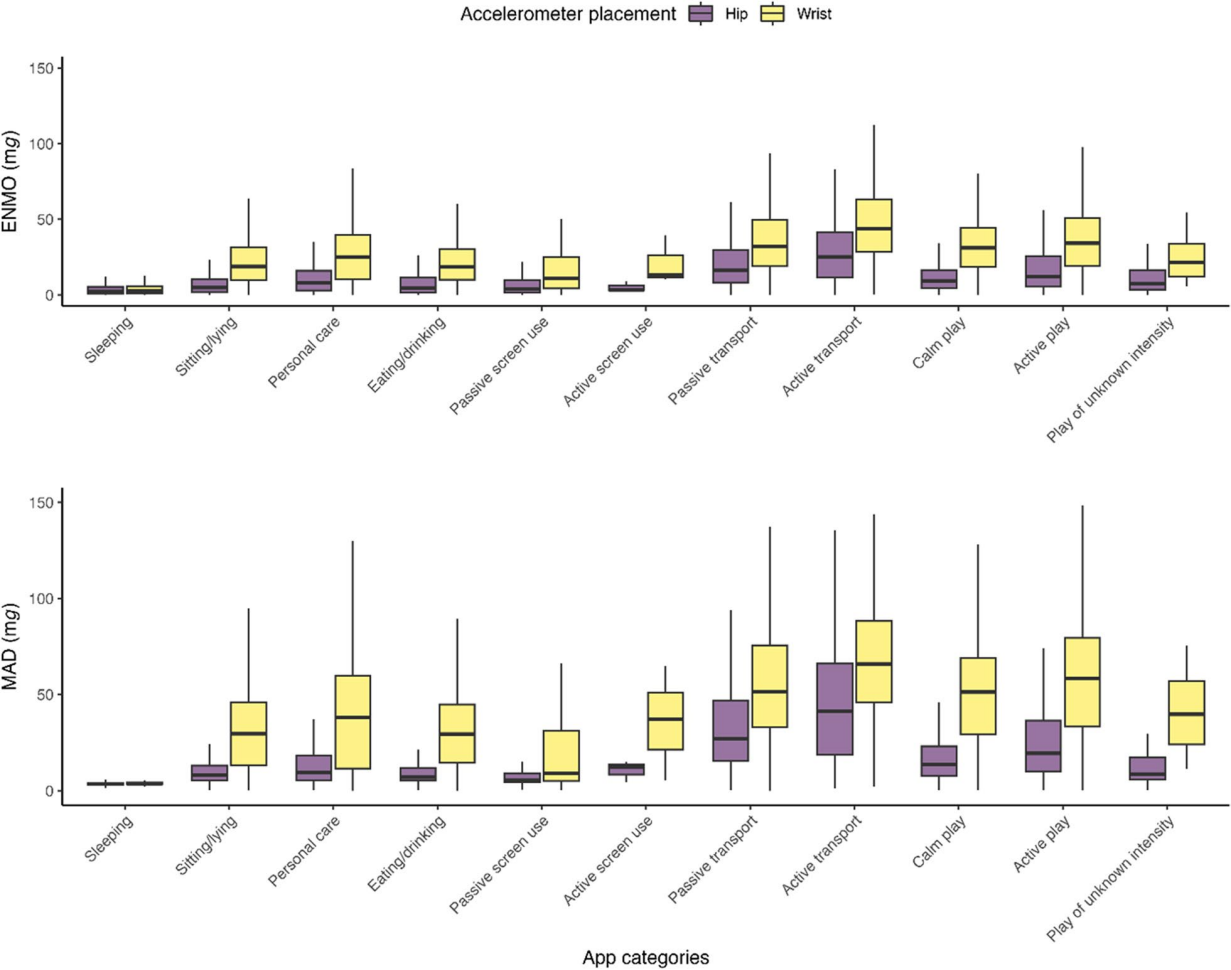
Euclidean norm minus one (ENMO) and mean amplitude deviation (MAD) acceleration of both hip and wrist accelerometers for all activity categories assessed by the My Little Moves app

Figure 6 presents the distribution of acceleration in the different activity categories. Regarding the hypothesized similarities between the app-based SB activity categories in acceleration, we found consistent support for 2 out of 21 sub-hypotheses, inconsistent support for 9 out of 21, and no support for 11 out of 21 (see Table 4). As hypothesized, across both placements and both metrics, acceleration did not significantly differ between the categories sitting/lying and eating/drinking (*p* = .130–.671), and between calm play and play of unknown intensity (*p* = .071–.824).

Contrary to our hypotheses, acceleration was significantly higher across both placements and both metrics during passive transport than during sitting/lying, personal care, eating/drinking, and passive screen use. Additionally, during calm play, we found significantly higher acceleration than during sitting/lying, eating/drinking, and passive screen use. Concerning passive screen use, we found significantly lower acceleration when compared to sitting/lying, personal care, eating/drinking.

#### Sub-hypotheses 22–24: similarities in accelerometer-derived acceleration across app-based activity categories classified as PA

We found inconsistent support for all three sub-hypotheses regarding similarities in acceleration across the app-based PA activity categories (Table 4). Contrary to our hypotheses, hip acceleration (for both ENMO and MAD) was significantly higher during active transport compared to active play and active screen use. Additionally, hip acceleration (for MAD) was significantly lower during active screen use compared to active play.

#### Sub-hypothesis 25–55: differences in accelerometer-derived acceleration between app-based activity categories classified as SB, PA and sleep

For the hypothesized differences in acceleration between app-based SB, PA and sleep activity categories, we found consistent support for 19 out of 31 sub-hypotheses, inconsistent support for 4 out of 31, and no support for 8 out of 31 sub-hypotheses (Table 4). As expected, our findings indicate significantly lower acceleration during sleeping compared to other activity categories, except for hip acceleration during active screen use (ENMO *p* = .782, MAD *p* = .099). In addition, acceleration during active transport and active play was significantly higher when compared to sitting/lying, personal care, eating/drinking, passive screen use and calm play.

Contrary to our hypotheses, acceleration during active screen use was not significantly higher than during SB activities including passive screen use (*p* = .146–.895), sitting/lying (*p* = .472–.788), personal care (*p* = .264–.868), eating/drinking (*p* = .571–.918), calm play (*p* = .218–.764) and play of unknown intensity (*p* = .282–.764). Also, hip acceleration was significantly higher during passive transport compared to active play, while wrist placement values were similar for both activities (ENMO *p* = .333, MAD *p* = .665, respectively).

## Discussion

In this study, we aimed to determine the minimum reporting time needed for reliable assessment of 24-h movement behaviors of children aged 0–4 years using the MLM app. We found that for the MLM app at least 2 days of 23 h were required to assess the composition of 24-h movement behaviors. In addition, to gain insight into the ability of the MLM app to assess 24-h movement behaviors in young children, we tested several hypotheses regarding differences between or similarities across app-based outcomes and acceleration data. As expected, acceleration was lowest during sleep, followed by SB, and highest during PA. When comparing activity categories, we found consistent support for 21 out of 55 sub-hypotheses. In general, we observed significantly higher acceleration for active play and active transport than for sedentary activities categories, except for passive transport.

Based on the results of our study, parents are required to complete the MLM app for a minimum of two to four days, depending on the outcome of interest. Further extending the reporting time yields more reliable data on children’s 24-h movement behaviors, which necessitates finding a balance between reliability and the feasibility of using the app over longer periods. Although the majority of parents who used the MLM app were able to fulfill the reporting requirements without additional compensation (i.e., 83.3% completed the app for at least 2 days, and 66.7% for at least 4 days), it is important to acknowledge that this may not be the case for all families, and considerations for the practicality and potential challenges of implementing the MLM app should be taken into account. Especially, given the difficulty in recruiting participants for our study, and the large drop-out because parents did not report any activities in the MLM app (22.0%), the use of such an app appears to be a barrier for many parents. Unfortunately, it is challenging to compare the feasibility with other proxy-report tools for this age group, as the feasibility of these tools has rarely been investigated [15, 49]. Additionally, young children often spend significant periods of the day outside their parents' supervision, such as in childcare, leading to missing data and possibly a systematic bias due to differences in 24-h movement behaviors during versus outside childcare [50, 51]. Unfortunately, we did not collect data on the frequency or time participating children spent in formal and/or informal childcare settings.

Although our hypotheses on differences in acceleration between the app-based 24-h movement behaviors were confirmed, the comparison of some specific activity categories did not align with our expectations. Specifically, we found no support for 19 out of 55 sub-hypotheses on similarities and differences in acceleration between specific activity categories. Notably, during passive transport acceleration was significantly higher than during other sedentary activities, and not different from acceleration during physical activities. This finding clearly shows a limitation of current accelerometer analysis methods, namely that these do not take into account that accelerometer output in young children may reflect the movement of others, such as parents pushing their child in a stroller. This further indicates the need for exploring novel accelerometer data processing approaches to improve accuracy in assessment of young children's 24-h movement behaviors. Alternatively, to examine validity of specific postures or activity categories, video-recorded direct observation of young children’s activities could be explored [52, 53]. In addition, as might be expected, acceleration during calm play was higher than during most sedentary activities (i.e., sitting/lying, eating/drinking, and passive screen use) but lower than during active play and active transport. This suggests the need for more specific activity intensities in the assessment of PA using the MLM app, such as light PA and moderate-to-vigorous PA. Unfortunately, the classification of intensity levels for physical activities in early childhood is hindered by the absence of corresponding metabolic equivalents, and clear definitions of PA in infants, toddlers and preschoolers [54]. For this reason, establishing guidelines for defining PA and SB in early childhood has been identified as a research priority [55]. Moreover, we were not able to confirm multiple hypotheses regarding screen use. For example, acceleration during passive screen use was lower than during other sedentary activities (i.e., sitting/lying, eating/drinking and personal care), which suggests that children are most sedentary during passive screen use. This aligns with the conclusions drawn in a previous laboratory study among children aged 10 to 12 and adolescents aged 16 to 18 years old, indicating that screen-based sedentary activities involved less body movement than non-screen-based sedentary activities [56]. In addition, we did not find significant differences in acceleration between active and passive screen use, but the low number of reported active screen use (three times) hampers our ability to draw conclusions for this activity.

Similarly, although for the follow-up question on posture, “changing posture” was commonly selected, some specific postures such as “lying on the tummy” were rarely reported. This can be explained by parents typically reporting activities in blocks of around 30 min. Consequently, the MLM app is less sensitive to capture rapid and sporadic posture changes, limiting the ability to assess frequency and duration of time spent in specific postures and, therefore, to monitor adherence to tummy time recommendations in infants specifically [57]. Incorporating more sensitive measurement instruments, such as accelerometers to develop posture classification algorithms, could enhance our ability to assess and analyze 24-h movement behaviors of young children more accurately.

Our findings suggest that 24-h movement behaviors reported in the app correspond to periods of high (PA), lower (SB), and lowest (sleep) acceleration, and therefore provide preliminary evidence for construct validity of the app. Stronger evidence for construct validity of the MLM app requires comparison between the time-stamped accelerations and the time-stamped app data to evaluate time concordance between the constructs sleep, SB and PA [58]. However, there are no validated cut-points or algorithms for translating acceleration into sleep, SB and PA for the full age range (0–4 years) of our sample [9], which limited our ability to assess time concordance. While promising developments have been made for classifying SB and PA in toddlers and preschoolers [9, 59, 60], such models for infants are currently lacking. Therefore, future studies are recommended to develop data-driven models to translate accelerometer data into movement behavior estimates, to reduce current gaps in evidence for young children [61]. Such models should also include daytime napping in young children [62].

Recently, two other proxy-report tools have been developed to assess 24-h movement behaviors in children aged 0–5 years: the “Movement Behaviour Questionnaire Baby” (MBQ-B) for infants and toddlers who have not yet reached their walking milestone, and the child version (MBQ-C) for toddlers and preschoolers who have achieved their walking milestone [63, 64]. These recall questionnaires demonstrated acceptable test–retest reliability and validity, showing promise for monitoring 24-h movement behaviors in these age groups [63]. Short-form questionnaires such as the MBQ offer advantages in terms of feasibility for parents. However, the MLM app enables real-time reporting and provides a more complete picture of all activities performed during a 24-h day, which allows for a comprehensive evaluation of the composition of 24-h movement behaviors.

## Strengths and limitations

Strengths of this study include the comprehensive assessment of minimum reporting requirements to obtain reliable proxy-report data in young children. Another strength is the measurement with both hip- and wrist-worn accelerometers. Also, although validity of accelerometers is yet to be established in infants and toddlers [9], we obtained valuable insights into the ability of the MLM app in assessing 24-h movement behaviors in young children by comparing app-based outcomes with acceleration data based on hypothesis testing.

Despite these strengths, our study has several limitations. One limitation is our app-based classification of activities solely into PA or SB, without considering different PA intensities such as light versus moderate intensity. Additionally, the activity categories “I don’t know”, “my child was with someone else”, and “other activity” were excluded in our analysis which may have resulted in a systematic bias. Another limitation is the potential error introduced by matching 5-min interval parent-reported activities with accelerometer data processed in 5-s epochs, leading to possible discrepancies due to rounding and timing inaccuracies, affecting the precision of hypotheses testing. Also, the majority of parents were female and highly educated, which limits the generalizability of our findings. Furthermore, although the sample size of participants with both app and accelerometer data can be considered adequate, it falls below the recommended threshold for studies evaluating measurement instruments, typically requiring at least 100 participants for a "very good" score [65]. Moreover, it must be recognized that we examined the minimum reporting time for reliable data over a single measurement period of 2 to 7 days, which may not accurately reflect young children’s habitual 24-h movement behaviors over a longer period, including differences due to seasonal variations or developmental changes [66]. Finally, some activity categories and postures in the app were rarely selected by parents, which limits our ability to draw conclusions regarding their classification into specific intensities.

## Recommendations for future studies

We recommend future studies to delve deeper into the classification of PA and SB based on activity categories and follow-up questions on intensity and posture in the MLM app, to possibly improve the app’s accuracy in 24-h movement behavior assessment. Moreover, given the wide variability in compliance, we recommend that future studies consider the impact of different reporting criteria on feasibility, and consequently the study sample size. Furthermore, recognizing the limitations of both proxy-report tools and accelerometers in accurately assessing 24-h movement behaviors, we recommend future studies to consider the combined use of accelerometers alongside proxy-report tools like the MLM app. By leveraging the advantages of both instruments, researchers can potentially obtain complementary data, thereby enhancing the overall validity of the 24-h movement behavior assessment [49]. Last, we encourage future studies to explore more advanced accelerometer data processing approaches aimed at accurately estimating specific postures and activities such as passive transport.

## Conclusions

The MLM app can be used to obtain reliable data on 24-h movement behaviors in 0–4-year-old children, provided that activities are reported for a minimum of 2 days of at least 23 h. The MLM app is promising for assessment of 24-h movement behaviors, although some specific activities and postures require further investigation. For example, exploring novel accelerometer data processing approaches will contribute to a more nuanced assessment of specific activities and better understanding of young children's 24-h movement behaviors.

## Supplementary Information

Supplementary Material 1.

Supplementary Material 2.

## Data Availability

The R scripts used for analyses of data from this study are available in our GitHub repository (https://zenodo.org/records/14965770). Data that support the findings of the study are available from the corresponding author upon reasonable request.

## Abbreviations

App: Application
BMI: Body mass index
ECEC: Early childhood education and care
ENMO: Euclidean norm minus one
GLMMs: General linear mixed-effects models
ICCs: Intra-class correlations
ilr: Isometric log-ratio
MAD: Mean amplitude deviation
MBQ-B: Movement Behaviour Questionnaire Baby
MBQ-B: Movement Behaviour Questionnaire Child
MLM: My Little Moves
NA: Not applicable
PA: Physical activity
SB: Sedentary behavior
WHO: World Health Organization
