# Supplementary material for: Assessing 24-h movement behaviors in early childhood (0–4 years): Reliability of the My Little Moves app and comparison with accelerometry
Source: J Act Sedentary Sleep Behav. 2025 Apr 7;4:5. doi: 10.1186/s44167-025-00075-x (PMC11974202; doi:10.1186/s44167-025-00075-x)
Supplement: Supplementary file 1 — Supplementary Material 1. [file 44167_2025_75_MOESM1_ESM.pdf]

STROBE Statement—Checklist of items that should be included in reports of ***cross-sectional studies***

	Item No	Recommendation	Page No
**Title and abstract**	1	(*a*) Indicate the study's design with a commonly used term in the title or the abstract	1—2
		(*b*) Provide in the abstract an informative and balanced summary of what was done and what was found	2
**Introduction**			
Background/rationale	2	Explain the scientific background and rationale for the investigation being reported	3—4
Objectives	3	State specific objectives, including any prespecified hypotheses	4
**Methods**			
Study design	4	Present key elements of study design early in the paper	4—5
Setting	5	Describe the setting, locations, and relevant dates, including periods of recruitment, exposure, follow-up, and data collection	5—8
Participants	6	(*a*) Give the eligibility criteria, and the sources and methods of selection of participants	4—5
Variables	7	Clearly define all outcomes, exposures, predictors, potential confounders, and effect modifiers. Give diagnostic criteria, if applicable	8—11
Data sources/ measurement	8*	For each variable of interest, give sources of data and details of methods of assessment (measurement). Describe comparability of assessment methods if there is more than one group	8—11
Bias	9	Describe any efforts to address potential sources of bias	12
Study size	10	Explain how the study size was arrived at	4—5
Quantitative variables	11	Explain how quantitative variables were handled in the analyses. If applicable, describe which groupings were chosen and why	8—11
Statistical methods	12	(*a*) Describe all statistical methods, including those used to control for confounding	12—14
		(*b*) Describe any methods used to examine subgroups and interactions	NA
		(*c*) Explain how missing data were addressed	8—10
		(*d*) If applicable, describe analytical methods taking account of sampling strategy	12
		(*e*) Describe any sensitivity analyses	NA
**Results**			
Participants	13*	(a) Report numbers of individuals at each stage of study—eg numbers potentially eligible, examined for eligibility, confirmed eligible, included in the study, completing follow-up, and analysed	15—16
		(b) Give reasons for non-participation at each stage	15—16
		(c) Consider use of a flow diagram	15
Descriptive data	14*	(a) Give characteristics of study participants (eg demographic, clinical, social) and information on exposures and potential confounders	15—16
		(b) Indicate number of participants with missing data for each variable of interest	15—16
Outcome data	15*	Report numbers of outcome events or summary measures	19—20

Main results	16	(*a*) Give unadjusted estimates and, if applicable, confounder-adjusted estimates and their precision (eg, 95% confidence interval). Make clear which confounders were adjusted for and why they were included	16—24<br><br>Additional file 2
		(*b*) Report category boundaries when continuous variables were categorized	Additional file 2
		(*c*) If relevant, consider translating estimates of relative risk into absolute risk for a meaningful time period	NA
Other analyses	17	Report other analyses done—eg analyses of subgroups and interactions, and sensitivity analyses	16—24<br>Additional file 2
**Discussion**			
Key results	18	Summarise key results with reference to study objectives	24—25
Limitations	19	Discuss limitations of the study, taking into account sources of potential bias or imprecision. Discuss both direction and magnitude of any potential bias	27—28
Interpretation	20	Give a cautious overall interpretation of results considering objectives, limitations, multiplicity of analyses, results from similar studies, and other relevant evidence	29
Generalisability	21	Discuss the generalisability (external validity) of the study results	27—28
**Other information**			
Funding	22	Give the source of funding and the role of the funders for the present study and, if applicable, for the original study on which the present article is based	29

*Give information separately for exposed and unexposed groups.

Note: An Explanation and Elaboration article discusses each checklist item and gives methodological background and published examples of transparent reporting. The STROBE checklist is best used in conjunction with this article (freely available on the Web sites of PLoS Medicine at http://www.plosmedicine.org/, Annals of Internal Medicine at http://www.annals.org/, and Epidemiology at http://www.epidem.com/). Information on the STROBE Initiative is available at www.strobe-statement.org.
