# Supplementary material for: Assessing 24-h movement behaviors in early childhood (0–4 years): Reliability of the My Little Moves app and comparison with accelerometry
Source: J Act Sedentary Sleep Behav. 2025 Apr 7;4:5. doi: 10.1186/s44167-025-00075-x (PMC11974202; doi:10.1186/s44167-025-00075-x)
Supplement: Supplementary file 2 — Supplementary Material 2. [file 44167_2025_75_MOESM2_ESM.pdf]

Additional file 2: Reliability and hypotheses testing: General linear mixed-effect model results

Corresponding to the article titled:
Assessing 24-hour movement behaviors in early childhood (0–4 years) - Reliability and construct validity of the My Little Moves app

Annelinde Lettink[1,2,3*], Jelle Arts[1,2*], Jessica S. Gubbels[4], Teatske M. Altenburg[1,2,3], and Mai J.M Chinapaw[1,2,3]

[*]Annelinde Lettink and Jelle Arts contributed equally to this work.
[1]Amsterdam UMC location Vrije Universiteit Amsterdam, Public and Occupational Health, De Boelelaan 1117, Amsterdam, The Netherlands;
[2]Amsterdam Public Health, Health Behaviors & Chronic Diseases, Amsterdam, The Netherlands;
[3]Amsterdam Public Health, Methodology, Amsterdam, The Netherlands;
[4]Maastricht University, Department of Health Promotion, NUTRIM School of Nutrition and Translational Research in Metabolism, PO Box 616, 6200 MD Maastricht, The Netherlands.

January 13, 2025

This additional file presents the differences in estimates (i.e., min/day spent in physical activity, sedentary behavior, and sleep, and the compositions) between week and weekend days, and the complete model output of the four general linear mixed-effects models (GLMMs) to test the hypothesized differences in accelerometer-derived acceleration between app-based 24-hour movement behaviors and activity categories.

Contents

1 Week and weekend day differences **3**

2 MLM app-based 24-hour movement behaviors **5**
2.1 Hip placement 5
2.2 Wrist placement 5

3 Activity categories **8**
3.1 Hip placement 8
3.2 Wrist placement 8

1 Week and weekend day differences

For each hourly increment from 12 up to 24 hours, six seperate models were fitted to test differences between app-based time-use estimates (i.e., min/day physcial activity, sedentary behavior, and sleep) and the first pivot coordinates of the 3-part compositions (i.e., ilr.PA, ilr.SB, and ilr.sleep) for week and weekend days:

1. PA $\sim$ weekendday + sex + age + (1 | participant id)
2. SB $\sim$ weekendday + sex + age + (1 | participant id)
3. sleep $\sim$ weekendday + sex + age + (1 | participant id)
4. ilr.PA $\sim$ weekendday + sex + age + (1 | participant id)
5. ilr.SB $\sim$ weekendday + sex + age + (1 | participant id)
6. ilr.sleep $\sim$ weekendday + sex + age + (1 | participant id)

Table 1 presents the differences between week days and weekend days for each app-based estimate per hourly increment.

Table 1: Weekday versus weekend day for the My Little Moves app-based estimates

**Minimum reporting time (hours/ day)**	**Number of participants $\geq$ 2 days, incl. $\geq$ 1 weekend day**	**PA (min/day)**			**SB (min/day)**			**Sleep (min/day)**			**ilr.PA**			**ilr.SB**			**ilr.sleep**		
		**Week day**	**Weekend day**	**p-value**	**Week day**	**Weekend day**	**p-value**	**Week day**	**Weekend day**	**p-value**	**Week day**	**Weekend day**	**p-value**	**Week day**	**Weekend day**	**p-value**	**Weekday**	**Week end day**	**p-value**
$\geq$ 12	310	105.00 [35.00; 210.00]	210.00 [120.00; 295.00]	**<.001**	280.00 [180.00; 405.00]	380.00 [291.25; 465.00]	**<.001**	715.00 [660.00; 810.00]	772.05 [690.00; 850.00]	**<.001**	-1.84 (1.63)	-1.18 (1.09)	**<.001**	0.01 (0.84)	-0.21 (0.63)	**<.001**	0.59 (0.74)	0.27 (0.47)	**<.001**
$\geq$ 13	308	105.00 [35.00; 210.00]	210.00 [120.00; 295.00]	**<.001**	280.00 [180.00; 410.00]	380.00 [295.00; 465.00]	**<.001**	720.00 [665.00; 810.00]	775.00 [690.00; 850.00]	**<.001**	-1.83 (1.62)	-1.18 (1.09)	**<.001**	0.01 (0.84)	-0.20 (0.62)	**<.001**	0.58 (0.74)	0.27 (0.47)	**<.001**
$\geq$ 14	305	110.00 [40.00; 210.00]	210.00 [120.00; 295.00]	**<.001**	290.00 [190.00; 415.00]	385.00 [300.00; 468.75]	**<.001**	725.00 [670.00; 820.00]	775.00 [696.25; 850.00]	**<.001**	-1.78 (1.58)	-1.18 (1.08)	**<.001**	0.00 (0.82)	-0.20 (0.61)	**<.001**	0.56 (0.72)	0.27 (0.47)	**<.001**
$\geq$ 15	303	132.50 [60.00; 230.00]	210.00 [120.00; 295.00]	**<.001**	325.00 [220.00; 435.00]	385.00 [300.00; 470.00]	**<.001**	745.00 [685.00; 835.00]	775.00 [700.00; 850.00]	**.029**	-1.58 (1.41)	-1.17 (1.06)	**<.001**	-0.07 (0.75)	-0.21 (0.61)	**<.001**	0.46 (0.64)	0.27 (0.46)	**<.001**
$\geq$ 16	298	170.00 [90.00; 255.00]	210.00 [120.00; 298.75]	**.002**	365.00 [280.00; 465.00]	385.00 [300.00; 465.00]	**<.001**	785.00 [710.00; 855.00]	775.00 [701.25; 850.00]	.517	-1.34 (1.18)	-1.15 (1.02)	**<.001**	-0.15 (0.66)	-0.21 (0.59)	**.024**	0.35 (0.52)	0.26 (0.44)	**<.001**
$\geq$ 17	290	180.00 [105.00; 265.00]	212.50 [120.00; 300.00]	**.009**	375.00 [300.00; 475.00]	385.00 [300.00; 466.25]	.077	790.00 [715.00; 860.00]	777.50 [710.00; 851.25]	.072	-1.31 (1.16)	-1.14 (0.99)	**.003**	-0.15 (0.65)	-0.22 (0.58)	**.035**	0.34 (0.51)	0.26 (0.43)	**.002**
$\geq$ 18	280	180.00 [110.00; 270.00]	215.00 [130.00; 300.00]	.057	385.00 [303.75; 475.00]	390.00 [300.00; 470.00]	.319	795.00 [720.00; 860.00]	780.00 [715.00; 850.00]	**.024**	-1.27 (1.11)	-1.14 (1.00)	**.023**	-0.17 (0.63)	-0.22 (0.58)	.094	0.32 (0.49)	0.26 (0.43)	**.018**
$\geq$ 19	276	185.00 [117.50; 270.00]	215.00 [130.00; 300.00]	.197	385.00 [310.00; 480.00]	390.00 [305.00; 470.00]	.411	800.00 [725.00; 865.00]	785.00 [715.00; 855.00]	.068	-1.25 (1.08)	-1.14 (1.00)	.108	-0.18 (0.62)	-0.22 (0.58)	.255	0.31 (0.47)	0.26 (0.43)	.088
$\geq$ 20	268	190.00 [120.00; 270.00]	215.00 [135.00; 300.00]	.535	390.00 [315.00; 481.25]	390.00 [305.00; 472.50]	.884	805.00 [730.00; 865.00]	790.00 [715.00; 855.00]	**.001**	-1.22 (1.03)	-1.14 (1.01)	.240	-0.19 (0.60)	-0.22 (0.59)	.389	0.29 (0.45)	0.26 (0.44)	.223
$\geq$ 21	257	197.50 [120.00; 280.00]	225.00 [135.00; 305.00]	.916	395.00 [315.00; 490.00]	390.00 [305.00; 473.75]	.631	805.00 [730.00; 865.00]	790.00 [730.00; 860.00]	**.003**	-1.18 (1.00)	-1.13 (1.01)	.550	-0.21 (0.59)	-0.23 (0.59)	.661	0.28 (0.43)	0.26 (0.43)	.604
$\geq$ 22	247	210.00 [120.00; 280.00]	225.00 [137.50; 307.50]	.931	402.50 [316.25; 490.00]	390.00 [307.50; 477.50]	.668	805.00 [740.00; 870.00]	795.00 [730.00; 860.00]	**.001**	-1.16 (0.95)	-1.11 (0.97)	.541	-0.21 (0.57)	-0.24 (0.58)	.701	0.27 (0.41)	0.25 (0.41)	.594
$\geq$ 23	226	210.00 [125.00; 285.00]	230.00 [142.50; 310.00]	.316	405.00 [320.00; 495.00]	395.00 [312.50; 480.00]	.996	805.00 [730.00; 875.00]	795.00 [730.00; 857.50]	**.002**	-1.13 (0.86)	-1.12 (1.00)	.767	-0.23 (0.53)	-0.23 (0.59)	.623	0.25 (0.36)	0.25 (0.43)	.712
24	187	210.00 [130.00; 285.00]	225.00 [140.00; 315.00]	.226	405.00 [325.00; 480.00]	400.00 [321.25; 490.00]	.490	815.00 [745.00; 875.00]	795.00 [730.00; 860.00]	**<.001**	-1.12 (0.89)	-1.14 (1.05)	.509	-0.24 (0.53)	-0.22 (0.61)	.318	0.25 (0.38)	0.26 (0.45)	.592

Note: Values are presented as Median [Inter Quartile Range, $25^{th}; 75^{th}$] min/day spent in PA, SB and sleep, and Median (Standard Deviation) ilr.PA, ilr.SB, and ilr.sleep
Abbreviations: MLM app My Little Moves app, *PA* physical activity, *SB* sedentary behavior

2 MLM app-based 24-hour movement behaviors

Table 2 presents descriptive data on the duration, frequency, and accelerometer-derived acceleration for all app-based 24-hour movement behaviors.

2.1 Hip placement

Two separate models were fitted to test the hypothesized differences in hip acceleration between app-based 24-hour movement behaviors:

1. ENMO $\sim$ 24-hour movement behavior sex + age + (participant id | 24-hour movement behavior)
2. MAD $\sim$ 24-hour movement behavior + sex + age + (participant id | 24-hour movement behavior)

Model summary Table 3 presents the random effects. By adding participant id over 24-hour movement behavior to the model as nested random effect we assume that observations from the same participant across app-based 24-hour movement behaviors are more similar than observations from other participants. Table 4 presents the fixed effect estimates of the model.

2.2 Wrist placement

Two separate models were fitted to test the hypothesized differences in wrist acceleration between the app-based 24-hour movement behaviors:

1. ENMO $\sim$ 24-hour movement behavior + sex + age + (participant id | 24-hour movement behavior)
2. MAD $\sim$ 24-hour movement behavior + sex + age + (participant id | 24-hour movement behavior)

Model summary Table 5 presents the random effects. By adding participant id over 24-hour movement behavior to the model as nested random effect we assume that observations from the same participant across app-based 24-hour movement behaviors are more similar than observations from other participants. Table 6 presents the fixed effect estimates of the model.

Table 2: Duration, frequency and acceleration per movement behavior over all reported activities. Values are presented as Median [Inter Quartile Range, 25th – 75th percentile] if not otherwise specified.

			Accelerometer-derived acceleration			
			**Hip acceleration**		**Wrist acceleration**	
**Activity category**	**Duration (min)**	**Frequency (%)***	**ENMO (mg)**	**MAD (mg)**	**ENMO (mg)**	**MAD (mg)**
**Sleep**	220.00 [95.00 – 360.00]	1522 (16.80)	2.30 [0.70 – 5.30]	3.50 [3.00 – 4.10]	2.80 [1.10 – 5.90]	3.70 [3.30 – 4.20]
**SB**	25.00 [15.00 – 30.00]	5710 (62.90)	7.40 [2.60 – 16.40]	9.70 [5.80 – 20.80]	23.50 [11.70 – 39.10]	36.70 [17.40 – 58.90]
**PA**	30.00 [20.00 – 60.00]	1532 (16.90)	12.80 [5.60 – 27.50]	20.90 [10.20 – 39.90]	34.70 [19.50 – 51.70]	59.00 [33.98 – 81.48]
**NA**	240.00 [60.00 – 525.00]	307 (3.38)	7.15 [3.00 – 12.40]	9.40 [6.10 – 17.05]	21.20 [10.75 – 31.98]	34.00 [18.43 – 51.63]

Abbreviations: ENMO Euclidean norm minus one, *MAD* mean amplitude deviation, *NA* not applicable, *PA* physical activity, *SB* sedentary behavior.

Table 3: Random effects of the app-based 24-hour movement behaviors for the acceleration during hip placement

	**ENMO**					**MAD**				
**Random effects**	*Variance*	*SD*	*ICC*			*Variance*	*SD*	*ICC*		
Movement behavior:Participant id	$1.26e^{-07}$	0.0004	0.11			$7.88e^{-08}$	0.0003	0.13		
Participant id	$8.57e^{-08}$	0.0003	0.07			$9.12e^{-09}$	0.0001	0.02		
Residual	$9.50e^{-07}$	0.001				$5.17e^{-07}$	0.0007			
	Log likelihood	*AIC*	*BIC*	$R^2 total$	$R^2 fixed$	*Log likelihood*	*AIC*	*BIC*	$R^2 total$	$R^2 fixed$
**Model fit**	44340.19	-88644.37	-88588.39	0.30	0.14	46802.87	-93589.74	-93533.75	0.38	0.28

Note. 8084 observations; 219 Movement behavior:Participant id, 74 Participant id

Table 4: Fixed effects of the app-based 24-hour movement behaviors for the acceleration during hip placement

**Fixed effects**		**ENMO**				**MAD**			
		Estimate	*95% CI*	*t(df)*	*p*	*Estimate*	*95% CI*	*t(df)*	*p*
Intercept (reference = sleep)		-0.01	[-0.006; -0.006]	-52.48 (89.27)	<.001***	-0.006	[-0.006; -0.006]	-83.71 (103.07)	<.001***
Sleep	SB	0.001	[0.0007; 0.0008]	11.39 (124.69)	<.001***	0.001	[0.0009; 0.001]	18.88 (121.31)	<.001***
	PA	0.001	[0.001; 0.001]	17.23 (155.05)	<.001***	0.001	[0.001; 0.002]	27.05 (147.79)	<.001***
Intercept (reference = SB)		-0.01	[-0.005; -0.005]	-46.60 (83.86)	<.001***	-0.005	[-0.005; -0.005]	-70.82 (93.65)	<.001***
SB	PA	0.0005	[0.0004; 0.0005]	6.97 (125.96)	<.001***	0.001	[0.0005; 0.0006]	9.79 (122.52)	<.001***
Sex (reference = male)		0.0001	[0.00004; 0.0001]	0.50 (69.62)	.616	0.00002	[-0.00002; 0.00007]	0.42 (66.10)	.678
Age		0.00002	[0.00001; 0.00002]	4.66 (72.38)	<.001***	0.00001	[0.00001; 0.00001]	4.88 (68.99)	<.001***

Table 5: Random effects of the app-based 24-hour movement behaviors for the acceleration during wrist placement

	**ENMO**					**MAD**				
**Random effects**	*Variance*	*SD*	*ICC*			*Variance*	*SD*	*ICC*		
Movement behavior:Participant id	1.24e-07	0.0004	0.12			$7.32e^{-08}$	0.0003	0.09		
Participant id	$7.58e^{-08}$	0.0003	0.08			$3.73e^{-08}$	0.0002	0.04		
Residual	$8.24e^{-07}$	0.0009				$7.47e^{-07}$	0.0009			
	Log likelihood	*AIC*	*BIC*	$R^2 total$	$R^2 fixed$	*Log likelihood*	*AIC*	*BIC*	$R^2 total$	R^2 *fixed*
**Model fit**	36915.48	-73814.96	-73760.54	0.42	0.28	37274.09	-74532.19	-74477.77	0.45	0.37

Note. 6649 observations; 205 24-hour Movement behavior:Participant id, 70 Participant id

Table 6: Fixed effects of the 24-hour movement behaviors for the wrist placement

**Fixed effects**		**ENMO**				**MAD**			
		Estimate	*95% CI*	*t(df)*	*p*	*Estimate*	*95% CI*	*t(df)*	*p*
Intercept (reference = sleep)		-0.006	[-0.006; -0.006]	-49.40 (70.66)	<.001***	-0.005	[-0.006; -0.005]	-62.60 (58.09)	<.001***
Sleep	SB	0.001	[0.001; 0.002]	21.24 (98.60)	<.001***	0.002	[0.002; 0.002]	29.87 (92.14)	<.001***
	PA	0.002	[0.002; 0.002]	25.39 (120.63)	<.001***	0.002	[0.002; 0.002]	34.77 (119.88)	<.001***
Intercept (reference = SB)		-0.004	[-0.004; -0.004]	-36.99 (66.18)	<.001***	-0.004	[-0.004; -0.004]	-44.18 (52.65)	<.001***
SB	PA	0.0004	[0.0004; 0.0005]	5.99 (100.24)	<.001***	0.0005	[0.0004; 0.0005]	8.06 (93.69)	<.001***
Sex (reference = male)		0.00001	[-0.00007; 0.0001]	0.16 (54.01)	.872	0.00006	[-0.00001; 0.00009]	0.85 (42.19)	.395
Age		0.000008	[0.000004; 0.00001]	2.02 (54.80)	.044*	0.000006	[-0.000003; 0.0001]	2.05 (43.21)	<.040*

3 Activity categories

Table 7 presents descriptive data on the duration, frequency, and accelerometer-derived acceleration for all app-based activities and postures.

3.1 Hip placement

Two separate GLMMs were fitted to test the hypothesized differences between activity categories during hip acceleration:

1. ENMO $\sim$ activity category + sex + age + (participant id | activity category)
2. MAD $\sim$ activity category + sex + age + (participant id | activity category)

Model summary Table 8 presents the random effects. By adding participant id over activity category to the model as nested random effect we assume that observations from the same participant across activity categories are more similar than observations from other participants. Table 9 presents the fixed effect estimates of the model.

3.2 Wrist placement

Two separate models were fitted to test the hypothesized differences between the activity categories during wrist acceleration:

1. ENMO $\sim$ activity category + sex + age +(participant id | activity category)
2. MAD $\sim$ activity category + sex + age + (participant id | activity category)

Model summary Table 10 presents the random effects. By adding participant id over activity category to the model as nested random effect we assume that observations from the same participant across activity categories are more similar than observations from other participants. Table 11 presents the fixed effect estimates of the model.

Table 7: Duration, frequency and acceleration per reported activity category over all reported activities. Values are presented as Median [Inter Quartile Range, 25th – 75th percentile] if not otherwise specified.

Activity category	Duration (min)	Frequency (%)*	Accelerometer-derived acceleration: Hip acceleration ENMO (mg)	Hip acceleration MAD (mg)	Wrist acceleration ENMO (mg)	Wrist acceleration MAD (mg)
**Sleeping**	220.00 [95.00 – 360.00]	1522 (16.80)	2.30 [0.70 – 5.30]	3.50 [3.00 – 4.10]	2.80 [1.10 – 5.90]	3.70 [3.30 – 4.20]
**Sitting/lying**	30.00 [15.00 – 40.00]	648 (7.14)	4.80 [1.80 – 10.50]	8.20 [5.40 – 13.00]	18.80 [9.70 – 31.40]	29.60 [13.30 – 46.00]
**Being carried**	20.00 [15.00 – 30.00]	39 (6.02)	1.65 [0.00 -3.38]	8.65 [6.63 – 11.53]	17.10 [9.75 – 39.45]	30.40 [16.50 – 46.40]
**Lying on tummy**	30.00 [17.50 – 30.00]	11 (1.70)	7.25 [1.58 – 8.90]	10.60 [6.70 – 13.85]	10.00 [5.83 – 13.60]	17.60 [14.38 – 20.85]
**Lying on back**	30.00 [20.00 – 43.75]	66 (10.20)	5.40 [1.40 – 8.65]	8.25 [5.23 – 12.45]	18.80 [11.50 -27.70]	27.50 [14.50 – 39.50]
**Lying on side**	60.00 [30.00 – 90.00]	5 (0.77)	5.80 [5.20 – 8.10]	6.30 [5.20 – 7.50]	8.50 [8.40 – 9.40]	13.30 [5.80 – 14.30]
**Sitting without support**	20.00 [15.00 – 30.00]	9 (1.39)	5.15 [2.43 – 28.18]	9.45 [5.48 – 17.55]	19.70 [10.35 – 26.50]	39.50 [21.15 – 47.45]
**Sitting with support**	30.00 [25.00 – 38.75]	74 (11.40)	3.80 [1.08 – 8.88]	8.20 [5.43 – 12.23]	23.00 [11.30 – 32.50]	35.10 [19.70 – 44.40]
**Lying**	25.00 [15.00 – 40.00]	84 (13.00)	4.45 [2.75 – 7.48]	5.15 [4.50 – 7.90]	13.50 [6.65 – 21.73]	12.10 [5.28 – 26.45]
**Sitting**	20.00 [11.25 – 30.00]	166 (25.60)	5.45 [1.60 – 13.80]	8.25 [5.90 – 14.03]	20.70 [11.70 – 34.60]	34.70 [15.00 – 53.80]
**Changing posture**	30.00 [22.50 – 50.00]	175 (27.00)	5.50 [2.75 – 11.50]	9.30 [5.95 – 14.90]	19.90 [9.55 – 34.80]	32.30 [15.45 – 50.95]
**I don't know**	20.00 [15.00 – 30.00]	19 (2.93)	3.80 [2.60 – 12.50]	10.90 [8.50 – 18.30]	13.80 [10.60 – 28.45]	23.90 [9.43 – 46.78]
**Personal care**	15.00 [10.00 – 20.00]	1308 (14.40)	7.90 [2.90 – 16.08]	9.40 [5.53 – 18.30]	25.20 [10.20 – 40.15]	38.20 [11.80 – 60.70]
**Eating/drinking**	30.00 [15.00 – 30.00]	1795 (19.80)	4.70 [1.40 – 11.50]	7.00 [5.20 – 11.70]	18.60 [10.03 – 30.28]	29.60 [14.53 – 42.23]
**Passive screen use**	30.00 [20.00 – 45.00]	177 (1.95)	4.00 [1.50 – 10.40]	5.50 [4.60 – 8.90]	11.00 [4.20 – 25.50]	9.45 [5.10 – 37.78]
**Active screen use**	30.00 [25.00 – 47.50]	3 (0.03)	3.10 [3.00 – 6.00]	12.20 [8.35 – 13.55]	13.00 [11.60 – 26.20]	37.10 [21.30 – 50.95]
**Passive transport**	30.00 [15.00 – 40.00]	1004 (11.10)	16.50 [8.30 – 30.00]	27.20 [15.70 – 47.35]	32.20 [18.90 – 50.20]	52.10 [33.50 – 76.40]
**Active transport**	25.00 [11.25 – 40.00]	186 (2.05)	25.70 [11.73 – 42.08]	41.80 [19.25 – 66.50]	44.00 [28.65 – 64.25]	67.80 [46.30 – 93.30]
**Calm play**	30.00 [20.00 – 60.00]	803 (8.85)	9.35 [4.50 – 16.40]	13.80 [7.80 – 23.13]	31.20 [18.68 – 44.45]	51.40 [29.28 – 69.48]
**Being carried**	20.00 [13.75 – 30.00]	8 (1.00)	5.80 [3.05 – 9.45]	9.90 [6.85 – 14.20]	33.90 [24.60 – 40.65]	55.30 [34.65 – 67.85]
**Lying on tummy**	30.00	1 (0.13)	24.50	22.40	35.10	52.90
**Lying on back**	30.00 [30.00 – 37.50]	15 (1.87)	4.80 [1.95 – 8.20]	8.00 [6.80 – 13.20]	20.30 [12.38 – 35.73]	31.30 [19.56 – 44.68]
**Lying on side**	30.00 [30.00 – 37.50]	2 (0.25)	19.20 [19.20 – 19.20]	26.90 [26.90 -26.90]	23.30 [21.25 – 23.35]	25.60 [15.37 – 35.73]
**Sitting without support**	30.00 [20.00 – 30.00]	17 (2.12)	2.00 [0.10 – 5.10]	7.20 [6.45 – 10.30]	23.40 [10.33 – 28.58]	38.70 [10.45 – 47.38]
**Sitting with support**	22.50 [16.25 – 30.00]	18 (2.24)	4.50 [1.65 – 15.05]	9.80 [6.60 – 14.53]	28.40 [14.60 – 42.43]	44.00 [26.18 – 59.35]
**Standing without support**	30.00 [28.75 – 41.25]	12 (1.49)	11.20 [9.33 – 13.83]	21.90 [10.15 – 24.78]	36.10 [26.43 – 38.73]	62.70 [44.00 - 66.33]
**Standing with support**	25.00 [17.50 -67.50]	7 (0.87)	11.90 [5.00 – 28.55]	8.90 [8.25 – 11.45]	12.30 [5.10 – 19.60]	7.70 [6.05 – 37.45]
**Lying**	30.00 [15.00 – 31.25]	12 (1.49)	10.50 [8.90 -13.35]	18.00 [12.30 – 21.70]	39.30 [27.60 – 39.70]	59.60 [56.40 – 64.70]
**Sitting**	30.00 [15.00 -45.00]	184 (22.90)	7.40 [3.80 – 12.98]	11.50 [7.08 – 17.33]	29.10 [16.00 – 43.30]	50.00 [26.80 – 63.80]
**Standing**	30.00 [20.00 – 60.00]	61 (7.60)	8.10 [3.80 – 16.40]	16.30 [6.50 – 26.90]	33.10 [20.85 – 41.90]	48.40 [16.75 - 62.00]
**Changing posture**	40.00 [30.00 – 66.25]	448 (55.80)	11.20 [6.10 – 19.20]	16.20 [9.20 – 26.60]	34.40 [19.70 – 49.68]	56.0 [33.85 – 76.75]
**I don't know**	30.00 [30.00 -43.75]	18 (2.24)	6.70 [2.75 – 9.30]	7.70 [5.90 – 8.40]	24.80 [19.70 – 31.28]	43.20 [30.13 – 52.93]
**Active play**	35.00 [25.00 – 60.00]	1293 (14.30)	12.2 [5.48 – 25.75]	19.5 [10.10 – 36.83]	34.4 [19.33 – 51.40]	59.0 [33.83 – 81.10]
**Being carried**	-	0	-	-	-	-
**Lying on tummy**	30.00 [20.00 – 38.75]	58 (4.49)	7.50 [3.80 – 10.50]	11.10 [9.00 – 15.30]	24.10 [14.50 – 32.90]	36.40 [23.50 – 51.20]
**Lying on back**	30.00 [20.00 – 50.00]	49 (3.79)	6.70 [4.10 – 8.90]	11.00 [9.30 – 13.70]	26.60 [11.20 – 36.98]	32.90 [20.85 – 48.80]
**Lying on side**	-	0	-	-	-	-
**Sitting without support**	30.00 [23.75 – 35.00]	28 (1.55)	3.05 [1.38 – 4.68]	8.80 [6.95 – 9.90]	21.20 [14.70 – 24.80]	36.90 [28.70 – 41.33]
**Sitting with support**	30.00 [23.75 – 35.00]	20 (1.55)	4.70 [1.98 – 9.63]	12.10 [8.43 – 15.83]	46.00 [26.05 – 54.60]	55.60 [45.05 – 68.15]
**Standing without support**	30.00 [23.75 – 56.25]	104 (8.04)	17.80 [10.85 – 27.53]	32.40 [23.08 – 52.00]	40.20 [2.80 – 54.95]	69.60 [44.30 – 90.90]
**Standing with support**	27.50 [15.00 – 35.00]	22 (1.70)	18.80 [10.20 – 52.53]	23.40 [11.15 – 37.20]	36.30 [17.30 – 43.80]	63.80 [31.80 – 74.60]
**Lying**	15.00 [10.00 -30.00]	15 (1.16)	16.00 [10.15 – 27.30]	30.40 [18.30 – 36.10]	44.90 [35.25 – 58.13]	78.40 [64.30 – 97.30]
**Sitting**	25.00 [13.75 – 30.00]	12 (0.93)	11.70 [5.10 – 18.85]	17.50 [11.90 – 23.10]	41.10 [25.10 – 54.63]	62.00 [50.65 – 85.48]
**Standing**	35.00 [20.00 – 60.00]	134 (10.40)	26.00 [12.25 – 40.45]	42.90 [21.45 – 65.95]	62.20 [43.35 – 83.25]	83.20 [65.40 – 121.55]
**Changing posture**	40.00 [30.00 – 65.00]	846 (65.40)	12.30 [5.23 – 25.60]	19.60 [9.63 – 35.50]	34.70 [18.90 – 50.70]	60.00 [33.90 – 81.40]
**I don't know**	45.00 [35.00 – 55.00]	5 (0.39)	4.20 [3.60 – 14.70]	21.60 [10.10 – 26.40]	29.00 [23.30 – 48.60]	52.90 [44.20 – 78.30]
**Play of unknown intensity**	60.00 [30.00 – 75.00]	25 (0.28)	7.50 [3.50 – 16.40]	8.70 [5.90 – 17.20]	21.40 [12.23 – 33.85]	39.80 [24.05 – 57.00]
**Being carried**	-	0	-	-	-	-
**Lying on tummy**	-	0	-	-	-	-
**Lying on back**	-	0	-	-	-	-
**Lying on side**	-	0	-	-	-	-
**Sitting without support**	-	0	-	-	-	-
**Sitting with support**	-	0	-	-	-	-
**Standing without support**	-	0	-	-	-	-
**Standing with support**	-	0	-	-	-	-
**Lying**	20.00 [15.00 – 25.00]	2 (8.00)	3.00 [2.70 – 3.30]	6.65 [6.38 – 6.93]	6.40 [5.95 – 6.85]	12.40 [11.80 – 13.00]
**Sitting**	50.00 [40.00 – 60.00]	2 (8.00)	3.65 [2.23 – 5.08]	6.40 [5.65 – 7.15]	10.50 [10.50 – 10.50]	24.90 [24.90 – 24.90]
**Standing**	-	0	-	-	-	-
**Changing posture**	60.00 [30.00 – 85.00]	11 (44.00)	14.50 [6.20 – 27.15]	10.50 [5.05 – 16.50]	41.80 [31.15 – 49.35]	69.00 [48.60 – 71.20]
**I don't know**	67.50 [26.25 – 86.25]	10 (40.00)	7.15 [3.33 – 9.50]	10.20 [8.43 – 17.73]	19.70 [13.40 – 26.15]	33.90 [23.13 – 48.33]
**Other activity**	25.00 [20.00 – 75.00]	83 (0.92)	7.70 [2.73 – 16.13]	11.00 [5.43 – 24.03]	24.20 [10.50 – 38.20]	37.60 [17.70 - 55.90]
**My child was with someone else**	510.00 [255.00 – 550.00]	197 (2.17)	6.65 [3.30 – 11.75]	8.55 [6.20 - 14.63]	20.00 [11.15 – 29.95]	32.20 [18.90 - 50.00]
**I don't know**	40.00 [30.00 – 82.50]	27 (0.30)	7.05 [1.40 – 18.08]	12.40 [6.45 – 19.55]	23.00 [10.20 – 32.30]	35.50 [19.20 – 47.00]

* For postures this is presented as % within the app category.

Abbreviations: ENMO Euclidean norm minus one, *MAD* mean amplitude deviation.

Table 8: Random effects of the activity categories for the acceleration during hip placement

	ENMO					MAD				
**Random effects**	*Variance*	*SD*	*ICC*			*Variance*	*SD*	*ICC*		
Activity category:Participant id	0.10	0.31	.09			0.07	0.27	.15		
Participant id	0.11	0.34	.11			0.02	0.16	.05		
Residual	0.84	0.91	-			0.39	0.63	-		
	Log likelihood	*AIC*	*BIC*	$R^2 total$	$R^2 fixed$	*Log likelihood*	*AIC*	*BIC*	$R^2 total$	$R^2 fixed$
**Model fit**	-11458.19	22954.38	23087.99	0.36	0.20	-8377.57	16793.51	16926.76	0.51	0.39

Note. 8370 observations; 700 Activity category:Participant id, 74 Participant id

Table 9: Fixed effects of the app categories for the hip placement

Fixed effects		ENMO				MAD			
		Estimate	*95% CI*	*t(df)*	*p*	*Estimate*	*95% CI*	*t(df)*	*p*
Intercept (reference = sleeping)		-5.99	[-6.21; -5.77]	-53.35 (96.06)	<.001***	-5.56	[-5.69; -5.43]	-85.46 (123.94)	<.001***
Sleeping	Sitting/lying	0.56	[0.42; 0.71]	7.62 (524.71)	<.001***	0.77	[0.65; 0.88]	13.14 (502.96)	<.001***
	Personal care	0.79	[0.66; 0.92]	12.04 (407.01)	<.001***	0.88	[0.77; 0.98]	16.66 (404.41)	<.001***
	Eating/drinking	0.48	[0.35; 0.60]	7.57 (362.76)	<.001***	0.74	[0.64; 0.84]	14.55 (368.49)	<.001***
	Passive screen use	0.23	[0.02; 0.44]	2.15 (909.68)	.032*	0.46	[0.30; 0.62]	5.59 (791.42)	<.001***
	Active screen use	0.16	[-0.95; 1.26]	0.28 (6362.14)	.782	0.66	[-0.12; 1.44]	1.65 (5278.48)	.099
	Passive transport	1.39	[1.26; 1.52]	20.84 (439.94)	<.001***	1.71	[1.60; 1.81]	31.99 (426.88)	<.001***
	Active transport	1.56	[1.36; 1.76]	15.01 (932.40)	<.001***	1.89	[1.74; 2.05]	23.81 (799.77)	<.001***
	Calm play	0.85	[0.72; 0.99]	11.89 (508.89)	<.001***	1.09	[0.98; 1.20]	19.15 (487.05)	<.001***
	Active play	1.21	[1.08; 1.33]	18.41 (399.44)	<.001***	1.47	[1.37; 1.57]	27.85 (399.16)	<.001***
	Play of unknown intensity	0.80	[0.33; 1.23]	3.69 (2566.47)	<.001***	0.80	[0.49; 1.11]	5.01 (2070.88)	<.001***
Intercept (reference = sitting/lying)		-5.43	[-5.66; -5.19]	-46.00 (116.61)	<.001***	-4.79	[-4.93; -4.65]	-68.19 (165.77)	<.001***
Sitting/lying	Personal care	0.22	[0.08; 0.37]	2.97 (549.96)	.003**	0.11	[-0.01; 0.22]	1.82 (526.09)	.068
	Eating/drinking	-0.09	[-0.23; 0.06]	-1.19 (509.38)	.235	-0.02	[-0.14; 0.09]	-0.42 (492.11)	.671
	Passive screen use	-0.33	[-0.56; -0.11]	-2.90 (985.50)	.004**	-0.31	[-0.48; -0.31]	-3.51 (861.53)	.001**
	Active screen use	-0.41	[-1.52; 0.70]	-0.72 (6337.46)	.472	-0.11	[-0.89; 0.68]	-0.27 (5257.99)	.788
	Passive transport	0.83	[0.68; 0.98]	10.80 (586.32)	<.001***	0.94	[0.82; 1.06]	15.67 (550.58)	<.001***
	Active transport	1.00	[0.78; 1.21]	8.99 (1004.33)	<.001***	1.13	[0.96; 1.29]	13.33 (872.16)	<.001***
	Calm play	0.29	[0.13; 0.45]	3.58 (647.09)	<.001***	0.32	[0.20; 0.44]	5.08 (605.32)	<.001***
	Active play	0.64	[0.49; 0.79]	8.51 (542.93)	<.001***	0.70	[0.59; 0.82]	11.80 (520.88)	<.001***
	Play of unknown intensity	0.24	[-0.19; 0.67]	1.09 (2557.07)	.275	0.03	[-0.29; 0.35]	0.19 (2072.38)	.853
Intercept (reference = personal care)		-5.20	[-5.42; -4.98]	-45.90 (99.76)	<.001***	-4.68	[-4.81; -4.55]	-71.00 (131.17)	<.001***
Personal care	Eating/drinking	-0.31	[-0.44; -0.18]	-4.82 (390.50)	<.001***	-0.13	[-0.23; -0.03]	-2.55 (392.74)	.011*
	Passive screen use	-0.56	[-0.77; -0.34]	-5.11 (921.07)	<.001***	-0.41	[-0.58; -0.25]	-4.98 (803.58)	<.001***
	Active screen use	-0.63	[-1.74; 0.48]	-1.12 (6362.53)	.264	-0.22	[-1.00; 0.57]	-0.54 (5281.13)	.589
	Passive transport	0.60	[0.47; 0.74]	8.85(466.58)	<.001***	0.83	[0.73; 0.94]	15.33 (450.76)	<.001***
	Active transport	0.77	[0.57; 0.98]	7.38 (945.79)	<.001***	1.02	[0.86; 1.17]	12.70 (814.14)	<.001***
	Calm play	0.07	[-0.08; 0.21]	0.90 (530.41)	.366	0.21	[0.10; 0.33]	3.69 (508.07)	<.001***
	Active play	0.42	[0.29; 0.55]	6.25 (427.02)	<.001***	0.59	[0.49; 0.70]	11.05 (423.49)	<.001***
	Play of unknown intensity	0.02	[-0.41; 0.45]	0.08 (2573.61)	.937	-0.08	[-0.39; 0.23]	-0.49 (2085.29)	.624
Intercept (reference = eating/drinking)		-5.51	[-5.73; -5.29]	-49.29 (94.72)	<.001***	-4.82	[-4.94; -4.69]	-74.46 (121.43)	<.001***
Eating/drinking	Passive screen use	-0.24	[-0.45; -0.03]	-2.28 (898.33)	.023*	-0.28	[-0.44; -0.12]	-3.42 (783.51)	<.001***
	Active screen use	-0.32	[-1.43; 0.79]	-0.57 (6364.23)	.571	-0.08	[-0.87; 0.70]	-0.21 (5281.06)	.835
	Passive transport	0.91	[0.79; 1.04]	13.87 (423.57)	<.001***	0.97	[0.86; 1.07]	18.26 (415.34)	<.001***
	Active transport	1.08	[0.88; 1.29]	10.48 (917.47)	<.001***	1.15	[0.99; 1.30]	14.53 (789.69)	<.001***
	Calm play	0.38	[0.24; 0.52]	5.31 (490.35)	<.001***	0.35	[0.24; 0.46]	6.13 (474.26)	<.001***
	Active play	0.73	[0.60; 0.86]	11.28 (384.00)	<.001***	0.73	[0.62; 0.83]	13.91 (388.03)	<.001***
	Play of unknown intensity	0.33	[-0.10; 0.75]	1.51 (2561.19)	.132	0.05	[-0.26; 0.37]	0.34 (2068.05)	.732
Intercept (reference = passive screen use)		-5.76	[-6.04; -5.47]	-39.66 (253.90)	<.001***	-5.10	[-5.28; -4.91]	-53.91 (440.23)	<.001***
Passive screen use	Active screen use	-0.08	[-1.19; 1.04]	-0.13 (6219.36)	.895	0.20	[-0.60; 0.99]	0.49 (5125.40)	.625
	Passive transport	1.16	[0.94; 1.37]	10.58 (945.23)	<.001***	1.25	[1.08; 1.41]	14.90 (819.40)	<.001***
	Active transport	1.33	[1.07; 1.59]	9.92 (1184.54)	<.001***	1.43	[1.23; 1.63]	14.14 (999.75)	<.001***
	Calm play	0.62	[0.40; 0.84]	5.54 (962.87)	<.001***	0.63	[0.46; 0.79]	7.33 (840.22)	<.001***
	Active play	0.97	[0.76; 1.19]	8.94 (916.62)	<.001***	1.01	[0.84; 1.17]	12.09 (800.15)	<.001***
	Play of unknown intensity	0.57	[0.11; 1.03]	2.43 (2417.53)	.015*	0.34	[0.00; 0.67]	1.95 (1966.53)	.051
Intercept (reference = active screen use)		-5.83	[-6.95; -4.71]	-10.18 (6144.61)	<.001***	-4.90	[-5.69; -4.11]	-12.17 (5318.93)	<.001***
Active screen use	Passive transport	1.23	[0.13; 2.34]	2.18 (6362.20)	.029*	1.05	[0.26; 1.83]	2.62 (5279.75)	.009**
	Active transport	1.40	[0.29; 2.52]	2.46 (6239.36)	.014*	1.23	[0.44; 2.03]	3.05 (5152.47)	.002**
	Calm play	0.70	[-0.41; 1.81]	1.23 (6345.46)	.218	0.43	[-0.36; 1.21]	1.07 (5268.77)	.285
	Active play	1.05	[-0.06; 2.16]	1.86 (6355.20)	.063	0.81	[0.03; 1.59]	2.03 (5272.74)	.043*
	Play of unknown intensity	0.65	[-0.53; 1.83]	1.08 (5945.48)	.282	0.14	[-0.70; 0.98]	0.32 (4883.52)	.747
Intercept (reference = passive transport)		-4.60	[-4.82; -4.37]	-40.29 (102.75)	<.001***	-3.85	[-3.98; -3.72]	-57.77 (136.57)	<.001***
Passive transport	Active transport	0.17	[-0.04; 0.38]	1.60 (970.42)	.109	0.18	[0.03; 0.34]	2.28 (830.67)	.023*
	Calm play	-0.54	[-0.68; -0.39]	-7.24 (563.38)	<.001***	-0.62	[-0.74; -0.51]	-10.61 (530.63)	<.001***
	Active play	-0.18	[-0.32; -0.05]	-2.69 (458.79)	.007**	-0.24	[-0.35; -0.13]	-4.38 (444.73)	<.001***
	Play of unknown intensity	-0.59	[-1.02; -0.16]	-2.68 (2582.95)	.007**	-0.91	[-1.22; -0.60]	-5.70 (2088.28)	<.001***
Intercept (reference = active transport)		-4.43	[-4.71; -4.15]	-31.33 (232.25)	<.001***	-3.67	[-3.84; -3.49]	-40.26 (402.93)	<.001***
Active transport	Calm play	-0.71	[-0.92; -0.49]	-6.54 (983.89)	<.001***	-0.80	[-0.97; -0.64]	-9.75 (849.19)	<.001***
	Active play	-0.35	[-0.56; -0.15]	-3.36 (939.92)	<.001***	-0.42	[-0.58; -0.27]	-5.26 (810.50)	<.001***
	Play of unknown intensity	-0.76	[-1.21; -0.30]	-3.24 (2469.81)	.001**	-1.10	[-1.43; -0.76]	-6.41 (2007.63)	<.001***
Intercept (reference = calm play)		-5.14	[-5.37; -4.90]	-43.44 (117.91)	<.001***	-4.47	[-4.61; -4.33]	-63.53 (167.56)	<.001***
Calm play	Active play	0.35	[0.21; 0.50]	4.81 (529.04)	<.001***	0.38	[0.27; 0.50]	6.58 (506.31)	<.001***
	Play of unknown intensity	-0.05	[-0.48; 0.38]	-0.22 (2576.57)	.824	-0.29	[-0.61; -0.02]	-1.81 (2087.83)	.071
Intercept (reference = active play)		-4.78	[-5.00; -4.56]	-42.21 (99.62)	<.001***	-4.09	[-4.22; -3.96]	-61.94 (130.95)	<.001***
Active play	Play of unknown intensity	-0.40	[-0.83; 0.03]	-1.84 (2553.86)	.065	-0.67	[-0.99; -0.36]	-4.21 (2066.36)	<.001***
Sex (reference = male)		0.02	[-0.15; 0.19]	0.25 (72.65)	<.800	0.02	[-0.07; 0.11]	0.45 (69.22)	<.653
Age		0.02	[0.01; 0.03]	4.74 (75.19)	<.001***	0.009	[0.00; .01]	4.37 (71.88)	<.001***

Table 10: Random effects of the activity categories for acceleration during wrist placement

	ENMO					MAD				
**Random effects**	*Variance*	*SD*	*ICC*			*Variance*	*SD*	*ICC*		
Activity category : Participant id	0.10	0.31	0.10			0.09	0.29	0.11		
Participant id	0.12	0.34	0.12			0.06	0.24	0.07		
Residual	0.76	0.87	-			0.66	0.81	-		
	Log likelihood	*AIC*	*BIC*	$R^2 total$	$R^2 fixed$	*Log likelihood*	*AIC*	*BIC*	$R^2 total$	$R^2 fixed$
**Model fit**	-9102.89	18243.77	18373.67	0.46	0.31	-8621.85	17281.70	17411.60	0.51	0.41

Note. 6883 observations; 626 Activity category:Participant id, 70 Participant id

Table 11: Fixed effects of the activity categories for acceleration during wrist placement

**Fixed effects**		ENMO				MAD			
		Estimate	*95% CI*	*t(df)*	*p*	*Estimate*	*95% CI*	*t(df)*	*p*
Intercept (reference = sleeping)		-5.68	[-5.91; -5.45]	-48.27 (75.47)	<.001***	-5.48	[-5.66; -5.30]	-60.12 (66.51)	<.001***
Sleeping	Sitting/lying	1.33	[1.17; 1.48]	17.11 (397.53)	<.001***	1.51	[1.37; 1.65]	20.77 (370.39)	<.001***
	Personal care	1.43	[1.29; 1.56]	20.57 (318.03)	<.001***	1.59	[1.46; 1.72]	24.36 (296.04)	<.001***
	Eating/drinking	1.41	[1.28; 1.54]	21.09 (285.36)	<.001***	1.62	[1.50; 1.74]	25.78 (265.83)	<.001***
	Passive screen use	0.93	[0.70; 1.15]	8.01 (726.58)	<.001***	0.93	[0.72; 1.14]	8.57 (678.26)	<.001***
	Active screen use	1.57	[0.51; 2.63]	2.90 (4916.04)	.004**	1.67	[0.68; 2.66]	3.31 (4829.58)	<.001***
	Passive transport	1.87	[1.73; 2.01]	26.43 (345.99)	<.001***	2.15	[2.02; 2.28]	32.34 (321.79)	<.001***
	Active transport	2.07	[1.84; 2.30]	17.90 (704.28)	<.001***	2.33	[2.11; 2.54]	21.45 (657.57)	<.001***
	Calm play	1.73	[1.58; 1.88]	22.87 (391.43)	<.001***	1.96	[1.82; 2.10]	27.57 (364.37)	<.001***
	Active play	1.94	[1.80; 2.08]	27.80 (316.44)	<.001***	2.18	[2.05; 2.31]	33.22 (294.70)	<.001***
	Play of unknown intensity	1.80	[1.34; 2.26]	7.60 (1742.56)	<.001***	2.06	[1.63 2.50]	9.31 (1662.36)	<.001***
Intercept (reference = sitting/lying)		-4.36	[-4.60; -4.12]	-35.28 (90.55)	<.001***	-3.97	[-4.16; -3.78]	-40.68 (85.88)	<.001***
Sitting/lying	Personal care	0.10	[-0.05; 0.26]	1.28 (414.40)	.199	0.08	[-0.07; 0.22]	1.04 (385.98)	.297
	Eating/drinking	0.08	[-0.07; 0.23]	1.09 (384.71)	.277	0.11	[-0.03; 0.25]	1.51 (358.53)	.130
	Passive screen use	-0.40	[-0.64; -0.16]	-3.29 (776.49)	<.001***	-0.58	[-0.81; -0.36]	-5.13 (725.19)	<.001***
	Active screen use	0.24	[-0.82; 1.30]	0.45 (4892.52)	.654	0.16	[-0.83; 1.15]	0.32 (4804.88)	.750
	Passive transport	0.55	[0.39; 0.70]	6.80 (444.68)	<.001***	0.64	[0.49; 0.79]	8.50 (413.91)	<.001***
	Active transport	0.75	[0.51; 0.98]	6.12 (747.23)	<.001***	0.81	[0.59; 1.04]	7.13 (698.69)	<.001***
	Calm play	0.41	[0.24; 0.57]	4.79 (484.30)	<.001***	0.45	[0.29; 0.60]	5.64 (451.41)	<.001***
	Active play	0.62	[0.46; 0.77]	7.75 (413.41)	<.001***	0.67	[0.52; 0.82]	8.98 (385.31)	<.001***
	Play of unknown intensity	0.47	[0.00; 0.94]	1.98 (1736.60)	.048*	0.55	[0.11; 0.99]	2.45 (1655.34)	.014*
Intercept (reference = personal care)		-4.26	[-4.49; -4.02]	-35.77 (78.44)	<.001***	-3.89	[-4.07; -3.71]	-42.05 (70.35)	<.001***
Personal care	Eating/drinking	-0.02	[-0.15; 0.12]	-0.26 (304.94)	.794	0.03	[-0.09; 0.16]	0.50 (283.94)	.620
	Passive screen use	-0.50	[-0.73; -0.27]	-4.31 (732.66)	<.001***	-0.66	[-0.88; -0.45]	-6.06 (683.66)	<.001***
	Active screen use	0.14	[-0.92; 1.20]	0.26 (4914.78)	.794	0.08	[-0.91; 1.07]	0.17 (4828.52)	.868
	Passive transport	0.44	[0.30; 0.59]	6.13 (364.31)	<.001***	0.56	[0.43; 0.70]	8.27 (338.73)	<.001***
	Active transport	0.64	[0.42; 0.87]	5.52 (710.99)	<.001***	0.74	[0.52; 0.56]	6.74 (663.50)	<.001***
	Calm play	0.30	[0.15; 0.46]	3.94 (404.76)	<.001***	0.37	[0.23; 0.51]	5.12 (376.91)	<.001***
	Active play	0.51	[0.37; 0.65]	7.19 (335.63)	<.001***	0.59	[0.46; 0.72]	8.81 (312.48)	<.001***
	Play of unknown intensity	0.37	[-0.09; 0.84]	1.57 (1745.66)	.117	0.47	[0.04; 0.91]	2.13 (1666.63)	.033*
Intercept (reference = eating/drinking)		-4.27	[-4.51; -4.04]	-36.41 (74.54)	<.001***	-3.86	[-4.04; -3.68]	-42.53 (65.37)	<.001***
Eating/drinking	Passive screen use	-0.48	[-0.71; -0.26]	-4.21 (716.63)	<.001***	-0.69	[-0.90; -0.48]	-6.43 (668.48)	<.001***
	Active screen use	0.16	[-0.90; 1.22]	0.29 (4917.00)	.769	0.05	[-0.94; 1.04]	0.10 (4830.95)	.918
	Passive transport	0.46	[0.32; 0.60]	6.60 (332.68)	<.001***	0.53	[0.40; 0.66]	8.07 (309.41)	<.001***
	Active transport	0.66	[0.44; 0.89]	5.74 (692.46)	<.001***	0.70	[0.49; 0.92]	6.53 (646.38)	<.001***
	Calm play	0.32	[0.17; 0.47]	4.30 (376.07)	<.001***	0.34	[0.20; 0.48]	4.81 (350.24)	<.001***
	Active play	0.53	[0.40; 0.67]	7.71 (304.09)	<.001***	0.56	[0.43; 0.69]	8.63 (283.22)	<.001***
	Play of unknown intensity	0.39	[-0.087 0.85]	1.65 (1737.62)	.099	0.44	[0.01; 0.87]	1.99 (1657.62)	.046*
Intercept (reference = passive screen use)		-4.76	[-5.06; -4.46]	-30.90 (208.39)	<.001***	-4.55	[-4.81; -4.30]	-34.79 (247.08)	<.001***
Passive screen use	Active screen use	0.64	[-0.43; 1.72]	1.17 (4776.42)	.241	0.75	[-0.26; 1.75]	1.46 (4679.77)	.146
	Passive transport	0.95	[0.72; 1.18]	8.08 (752.58)	<.001***	1.22	[1.01; 1.44]	11.15 (701.88)	<.001***
	Active transport	1.15	[0.86; 1.43]	7.79 (905.23)	<.001***	1.40	[1.13; 1.67]	10.15 (847.59)	<.001***
	Calm play	0.81	[0.57; 1.04]	6.75 (757.95)	<.001***	1.03	[0.81; 1.25]	9.22 (707.87)	<.001***
	Active play	1.02	[0.79; 1.25]	8.71 (732.37)	<.001***	1.25	[1.04; 1.47]	11.46 (683.20)	<.001***
	Play of unknown intensity	0.87	[0.37; 1.38]	3.42 (1677.13)	<.001***	1.13	[0.67; 1.60]	4.75 (1599.22)	<.001***

Table 11 continued from previous page

**Fixed effects**		**ENMO**				**MAD**			
		Estimate	*95% CI*	*t(df)*	*p*	*Estimate*	*95% CI*	*t(df)*	*p*
Intercept (reference = active screen use)		-4.12	[-5.19; -3.04]	-7.49 (4790.25)	<.001***	-3.81	[-4.81; -2.81]	-7.47 (5007.97)	<.001***
Active screen use	Passive transport	0.30	[-0.76; 1.36]	0.56 (4915.68)	.575	0.48	[-0.51; 1.47]	0.95 (4828.91)	.343
	Active transport	0.50	[-0.57; 1.58]	0.92 (4748.62)	.359	0.65	[-0.35; 1.66]	1.27 (4654.65)	.202
	Calm play	0.16	[-0.90; 1.22]	0.30 (4899.58)	.764	0.29	[-0.70; 1.28]	0.57 (4813.81)	.571
	Active play	0.37	[-0.69; 1.43]	0.69 (4907.04)	.490	0.51	[-0.48; 1.50]	1.01 (4819.85)	.314
	Play of unknown intensity	0.23	[-0.92; 1.38]	0.40 (4392.64)	.693	0.39	[-0.68; 1.46]	0.71 (4294.94)	.478
Intercept (reference = passive transport)		-3.81	[-4.05; -3.58]	-31.81 (80.81)	<.001***	-3.33	[-3.51; -3.15]	-35.59 (73.42)	<.001***
Passive transport	Active transport	0.20	[-0.03; 0.43]	1.70 (732.12)	.089	0.17	[-0.04; 0.39]	1.58 (683.21)	.114
	Calm play	-0.14	[-0.29; 0.01]	-1.79 (432.66)	.074	-0.19	[-0.34; -0.05]	-2.61 (402.65)	.009**
	Active play	0.07	[-0.07; 0.21]	0.97 (363.10)	.333	0.03	[-0.10; 0.16]	0.43 (337.65)	.665
	Play of unknown intensity	-0.07	[-0.54; 0.40]	-0.30 (1752.52)	.765	-0.09	[-0.53 0.35]	-0.40 (1672.61)	.686
Intercept (reference = active transport)		-3.61	[-3.91; -3.31]	-23.64 (202.34)	<.001***	-3.15	[-3.41; -2.90]	-24.46 (238.72)	<.001***
Active transport	Calm play	-0.34	[-0.57; -0.11]	-2.84 (732.50)	.005**	-0.37	[-0.59; -0.15]	-3.27 (684.21)	<.001***
	Active play	-0.13	[-0.36; 0.10]	-1.10 (711.17)	.270	-0.14	[-0.36; 0.07]	-1.31 (663.91)	.189
	Play of unknown intensity	-0.27	[-0.77; 0.23]	-1.06 (1658.08)	.289	-0.26	[-0.73; 0.20]	-1.10 (1581.54)	.269
Intercept (reference = calm play)		-3.95	[-4.20; -3.71]	-31.85 (92.56)	<.001***	-3.52	[-3.71; -3.33]	-35.84 (88.64)	<.001***
Calm play	Active play	0.21	[0.06; 0.36]	2.71 (408.50)	.007**	0.22	[0.08; 0.36]	3.04 (380.27)	.002**
	Play of unknown intensity	0.07	[-0.40; 0.54]	0.29 (1749.37)	.774	0.10	[-0.34; 0.54]	0.46 (1667.97)	.648
Intercept (reference = active play)		-3.74	[-3.98; -3.51]	-31.41 (78.79)	<.001***	-3.30	[-3.48; -3.12]	-35.58 (70.62)	<.001***
Active play	Play of unknown intensity	-0.14	[-0.61; 0.32]	-0.60 (1732.80)	.551	-0.12	[-0.56; 0.32]	-0.54 (1653.67)	.590
Sex (reference = male)		-0.02	[-0.20; 0.17]	0.19 (57.41)	<.850	0.04	[-0.09; 0.18]	0.60 (43.65)	.549
Age		0.01	[0.00; 0.01]	2.37 (57.68)	<.018*	0.01	[0.00; 0.01]	2.39 (44.10)	.017**
